# Semiautomated glycoproteomics data analysis workflow for maximized glycopeptide identification and reliable quantification

**DOI:** 10.3762/bjoc.16.253

**Published:** 2020-12-11

**Authors:** Steffen Lippold, Arnoud H de Ru, Jan Nouta, Peter A van Veelen, Magnus Palmblad, Manfred Wuhrer, Noortje de Haan

**Affiliations:** 1 Center for Proteomics and Metabolomics, Leiden University Medical Center, Albinusdreef 2, 2333 ZA Leiden, Netherlands

**Keywords:** bioinformatics, cysteine oxidation, glycoproteomics, immunoglobulins, mass spectrometry

## Abstract

Glycoproteomic data are often very complex, reflecting the high structural diversity of peptide and glycan portions. The use of glycopeptide-centered glycoproteomics by mass spectrometry is rapidly evolving in many research areas, leading to a demand in reliable data analysis tools. In recent years, several bioinformatic tools were developed to facilitate and improve both the identification and quantification of glycopeptides. Here, a selection of these tools was combined and evaluated with the aim of establishing a robust glycopeptide detection and quantification workflow targeting enriched glycoproteins. For this purpose, a tryptic digest from affinity-purified immunoglobulins G and A was analyzed on a nano-reversed-phase liquid chromatography–tandem mass spectrometry platform with a high-resolution mass analyzer and higher-energy collisional dissociation fragmentation. Initial glycopeptide identification based on MS/MS data was aided by the Byonic software. Additional MS1-based glycopeptide identification relying on accurate mass and retention time differences using GlycopeptideGraphMS considerably expanded the set of confidently annotated glycopeptides. For glycopeptide quantification, the performance of LaCyTools was compared to Skyline, and GlycopeptideGraphMS. All quantification packages resulted in comparable glycosylation profiles but featured differences in terms of robustness and data quality control. Partial cysteine oxidation was identified as an unexpectedly abundant peptide modification and impaired the automated processing of several IgA glycopeptides. Finally, this study presents a semiautomated workflow for reliable glycoproteomic data analysis by the combination of software packages for MS/MS- and MS1-based glycopeptide identification as well as the integration of analyte quality control and quantification.

## Introduction

Protein glycosylation mainly occurs in the form of *N*- and *O*-glycosylation. *N*-Glycans are attached to Asn within an amino acid consensus sequence (Asn-Xxx-Ser/Thr, Xxx ≠ Pro) and *O*-glycans are attached to Ser or Thr. Glycan compositions can range from monosaccharides (e.g., Tn antigen for *O*-glycans [1]) to large polysaccharides (e.g., *N*-glycans of recombinant human erythropoietin [2]). The most common building blocks of human protein glycans are hexoses (glucose, galactose, and mannose, Hex/H, 162.0528 Da), *N*-Acetylhexosamines (*N*-acetylglucosamine or *N*-acetylgalactosamine, HexNAc/N, 203.0794 Da), fucose (Fuc/F, 145.0579 Da), and sialic acid (*N*-acetylneuraminic acid, NeuAc/S, 291.0954 Da). The combinatorial possibilities of these building blocks and the variety of structural features, such as the linkage position and anomeric configuration, make protein glycosylation a highly complex posttranslational modification (PTM).

Glycoproteomics has become important for many life science disciplines, in particular for biomedical and biopharmaceutical research [3–5]. Glycopeptide-centered glycoproteomics aims at the characterization of macroheterogeneity and microheterogeneity of protein glycosylation [6]. Reversed-phase liquid chromatography coupled to high-resolution tandem mass spectrometry (RPLC–MS/MS) is a standard analytical method in the field of glycoproteomics [7]. The separation of glycopeptides in RPLC is mainly driven by the peptide portions. Thus, information on different proteins and glycosylation sites appears in the form of glycopeptide clusters. Next to the peptide portion, glycosylation features, such as sialic acids, can strongly influence the retention time [8]. Advances in MS technologies tremendously enhanced the detection and informative fragmentation of glycopeptides in the past years [9]. The large amount of highly complex data acquired using these technologies shifted the major bottleneck in glycopeptide analysis to the data processing steps. Next to the high complexity of glycosylation itself, data analysis is further complicated by interfering background signals from biological matrices and isomeric and near-isobaric ambiguities resulting from combinations of monosaccharides, adducts, amino acids, and amino acid modifications [10–11].

Efforts have been made in recent years in the development of bioinformatic tools to facilitate and automate data processing in glycopeptide-centered glycoproteomics [12]. Several reports have reviewed the functionalities and application areas of data analysis tools in the field of glycoproteomics [7,9,12–13]. MS/MS-based scoring software tools such as Byonic [14] are frequently used for glycopeptide identification [12]. Recently, software tools were developed that are based on the retention time (RT) characteristics and accurate mass differences of glycopeptide MS1 signals in RPLC–MS [10,15]. These tools detect inaccuracies of MS/MS assignments based on the RT and increase the number of identified glycopeptide compositions while keeping the false positive assignments low. Other reports performed glycopeptide identification using summed MS1 spectra of previously defined elution clusters [16]. This approach is applicable when the identity and elution behavior of the glycopeptides of interest is known and is aided by quality criteria such as mass accuracy and isotopic pattern matching. Furthermore, such approaches allow quantification in a high-throughput manner, which is advantageous e.g., in clinal cohort analysis [16–18].

Here, we present a workflow for the reliable and efficient analysis of glycopeptides from enriched glycoproteins. We performed a thorough evaluation of the software tools and workflows used in our laboratory for the identification and quantification of glycopeptides. For this, a sample containing immunoglobulins G and A (IgG and IgA), simultaneously captured from human plasma, was chosen. This sample showed a considerable level of complexity due to the presence of multiple glycoproteins of interest and cocaptured (glyco)proteins from the plasma. The tools included Byonic, GlycopeptideGraphMS, Skyline, and LaCyTools.

## Results and Discussion

### Glycoproteomics data analysis workflow

**Figure 1:**
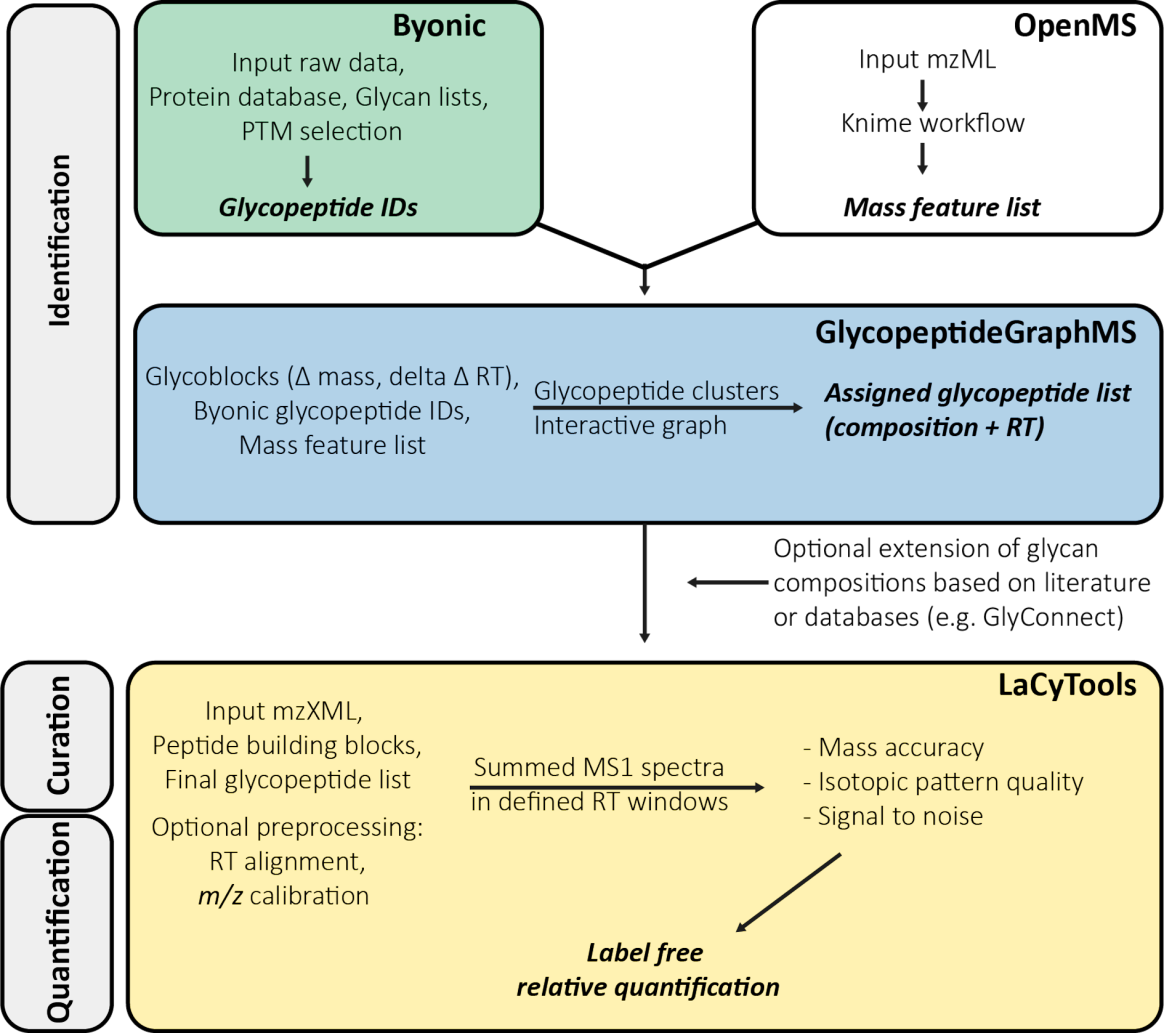
Integration of automated glycopeptide identification by Byonic and GlycopeptideGraphMS (aided by OpenMS) and subsequent analyte quality control and quantification by LaCyTools.

Affinity-copurified IgG/IgA from human plasma was chosen as a sample to demonstrate the integration of tools for the semiautomated glycoproteomic data analysis (Figure 1). The three main parts of this workflow cover glycopeptide identification (Byonic, GlycopeptideGraphMS), curation, and quantification (LaCyTools).

In the first step, Byonic was used for automated MS/MS-based (glyco)peptide identification. This initial step is crucial to validate the presence of glycopeptides and the assignment of the peptide portions. Next, the number of identified glycopeptides was maximized by performing an open search based on MS1 information (mass and RT) in GlycopeptideGraphMS. A preprocessing step in OpenMS was performed as described for the original GlycopeptideGraphMS workflow [15], including deisotoping and decharging of all features. The outcome of GlycopeptideGraphMS is a list with glycopeptide clusters (defined as LC–MS features (nodes) that are connected by Δmass and ΔRT within the provided limits for glycopeptides), for which at least one node should be confidently assigned by MS/MS to identify all glycopeptides in a cluster. The clusters are also presented in interactive graphs, which assist in the identification of false-positive connections (unlikely mass/RT shifts) and unexpected glycopeptide clusters (e.g., missed cleaved products and peptide or glycan modifications). This information can be used in an iterative manner to adjust the search space for Byonic. Study-specific search criteria are listed in the Experimental section and a detailed manual for the use of GlycopeptideGraphMS can be found elsewhere [15]. Of note, separate LC–MS runs with exclusively MS1 information were acquired in order to maximize the MS1-based identification and to ensure the highest possible data quality for the quantification purposes.

Upon glycopeptide identification, the list of glycopeptides generated by GlycopeptideGraphMS was transformed to the input format required for targeted curation and quantification in LaCyTools [16]. A python script was developed to facilitate this step (Supporting Information File 1). LaCyTools was chosen because it is open-source, can be applied for a large number of samples (thousands of samples in one study have been reported [19]), and allows data curation and quantification. Importantly, LaCyTools requires RT clusters to be defined in which MS1 spectra can be summed and further processed, which is facilitated by the GlycopeptideGraphMS output. The analyte list may be extended by including glycan compositions (e.g., from the literature or databases such as GlyConnect [20]) within appropriate RT clusters (e.g., the same peptide portion and number of sialic acids). Furthermore, the user has the option to perform preprocessing steps, such as *m*/*z* calibration and RT alignment. For data curation, summed MS1 spectra were subjected to quality control based on user-defined cut-offs for mass accuracy, isotopic pattern matching, and the signal-to-noise ratio of an analyte. Finally, the integrated areas of all charge states passing the quality criteria were summed for each glycopeptide composition, the area was corrected for missing isotopes, and total area normalization was performed for label-free relative quantification. Study-specific parameters for the use of LaCyTools are provided in the Experimental section and further explanation on the use of this tool can be found elsewhere [21].

### Glycopeptide identification

#### Automated MS/MS-based glycopeptide identification by Byonic

The automated and score-based MS/MS glycopeptide identification using Byonic resulted in the confident assignment of ten IgG/IgA *N*-glycopeptide clusters of interest (Table 1 and Figures S1–S10, Supporting Information File 2).

**Table 1:** Automated MS/MS-based identification of IgG/IgA glycosylation sites by Byonic. For each glycopeptide moiety, a representative glycoform is shown (see Figures S1–S10, Supporting Information File 2 for the corresponding MS/MS spectra).

Protein	Glycopeptide	Glycosylation site^a^	Cluster	Mass error (ppm)	Score	Scan time (min)

IgG1	R.EEQYN[+H5N4F1]STYR.V	Asn297	IgG1	0.7	589	14.4
IgG2/3	R.EEQFN[+H3N4F1]STFR.V	Asn297	IgG2/3	0	693	18.5
IgG4	R.EEQFN[+H3N4F1]STYR.V	Asn297	IgG4	1.1	401	15.8
IgA1/2	R.LSLHRPALEDLLLGSEAN[+H5N4S1]LTC[+57]TLTGLR.D	Asn263	LSL	0.9	839	40.2
	R.LAGKPTHVN[+H5N5F1S2]VSVVM[+16]AEVDGTC[+57]Y.-^b^	Asn459	LAGY	0.4	601	25.5
	R.LAGKPTHVN[+H5N5F1S2]VSVVM[+16]AEVDGTC[+57].-^b^	Asn459	LAGC	2.9	649	25.9
IgA2	K.TPLTAN[+H5N4F1S1]ITK.S	Asn337	TPL	−1.2	728	19.1
	K.HYTN[+H5N5F1S1]SSQDVTVPC[+57]R.V	Asn211	HYT	1.3	194	15.6
JC	R.EN[+H5N4S2]ISDPTSPLR.T	Asn49	ENI	0.1	565	22.2
	R.IIVPLNNREN[+H5N4F1S1]ISDPTSPLR.T	Asn49	IIV	1.2	271	28.0

^a^Numbering according to [18]. ^b^C-terminal peptide of the heavy chain, no C-terminal tryptic cleavage.

Assigned glycopeptides from copurified human plasma proteins other than IgG and IgA were not considered for further data processing (e.g., fibrinogen, alpha-1-antitrypsin, or clusterin, see Table S1A–E, Supporting Information File 3). Missed-cleavage variants were assigned for IgG1, IgG2/3, and IgA1/2 (Asn263) but not further considered because of their low abundance. For the IgA joining chain (JC), the elongated peptide with a missed cleavage was included for further data processing as the cleavage efficiency was previously determined to be glycoform dependent [18,22]. For the assignment of tryptic *N*-glycopeptides to specific proteins, ambiguities exist for one peptide moiety that could be assigned to either IgG2 or IgG3 and three moieties that were shared between IgA1 and IgA2 (Table 1) [3]. These ambiguities were not resolved using the proposed workflow. However, the presence of protein-specific (non)glycopeptides may indicate differences in the abundance of the individual proteins. For addressing these ambiguities, a more selective sample preparation is required, for example, using different enrichment strategies or proteases [23]. Interestingly, an additional allotype of the main IgG3 glycosylation site (EEQYNSTFR) was assigned in four out of five technical replicates by Byonic. This IgG3 glycopeptide is an isomer of the tryptic IgG4 glycopeptide (EEQFNSTYR). However, upon manual inspection of the data, only one scan of the assigned MS/MS spectra within all five technical replicates covered the relevant amino acids (position of Phe and Tyr), allowing an unambiguous discrimination between IgG3 or IgG4 (score 281, Figure S11, Supporting Information File 2). The IgA2 HYT glycopeptides had the lowest scores (max. 194) compared to the other glycosylation sites. It was detected in four out of five technical replicates and only with a maximum of one glycan composition. The low intensity of these glycopeptide signals resulted in a decreased likelihood for MS/MS selection. Of note, the IgA2 HYT glycopeptide covers a sequence stretch homologous to the hinge region of IgA1, carrying *O*-glycans. In a previous study the IgA1 peptide has been referred to as the HYT glycopeptide cluster as well [17]. The C-terminal IgA1/2 glycopeptides (LAGC/Y) were found mainly with methionine oxidation. Unoxidized peptide moieties were also assigned but with low scores (below 50). The manual check of the data revealed that in some cases, the selection of the wrong monoisotopic mass in Byonic led to misassignments of near-isobaric compositions, e.g., TPL H5N5F3 (3+, *m*/*z* 1074.8020, false) instead of TPL H5N5F1S1 (3+, *m*/*z* 1074.4619, correct). Other theoretical possible, but less common, tryptic IgG3, IgA1, and IgA2 glycopeptides were not detected [3,17]. One of the reported common miscleaved IgA2 *N*-glycopeptides (SESGQNVTAR) is likely to elute prior to MS acquisition as described previously for the applied gradient [17]. For the expected IgA1 *O*-glycopeptide cluster, the Byonic search failed to score any hits when performed as described previously [17]. Of note, the tryptic *O*-glycopeptide cluster could be detected upon manual inspection, albeit with low intensity (Figures S12 and S13, Supporting Information File 2). The reason for this was further investigated based on the GlycopeptideGraphMS results and is discussed in the section on automated MS1- and RT-based glycopeptide identification by GlycopeptideGraphMS.

In MS/MS scoring approaches such as Byonic, the definition of a threshold for the automated assignment of glycopeptides is generally a challenge as the scores depend largely on the fragmentation method, the peptide characteristics (e.g., peptide length or additional modifications), the glycome, and the sample matrix [11]. A recent study by us applied a threshold score of 200 for the IgG/IgA glycopeptides from human serum, aiming to find a balance between the exclusion of false positives while preventing false negatives [17]. Sensitive glycopeptide assignments relying only on oxonium ions and precursor mass, using a score above 30 were also described recently [15]. A suitable cut-off score should always be carefully evaluated for each (glyco)peptide moiety with respect to the glycoform coverage and accuracy [11].

Byonic identified the relevant *N*-glycosylation sites of IgG/IgA in all five technical replicates with the exception of the low-abundant IgA2 HYT glycopeptide. Further results and discussion of the accuracy and coverage of the investigated glycopeptides of interest are presented in the following section. Software tools for automated MS/MS-based assignments such as Byonic are highly useful in glycoproteomic data processing workflows. Other, noncommercial, automated MS/MS-based software tools for glycopeptide identification were recently reviewed [12] and have the potential to substitute Byonic in similar workflows as described here. However, these tools were not evaluated in the current study.

#### Automated MS1- and RT-based glycopeptide identification by GlycopeptideGraphMS

**Figure 2:**
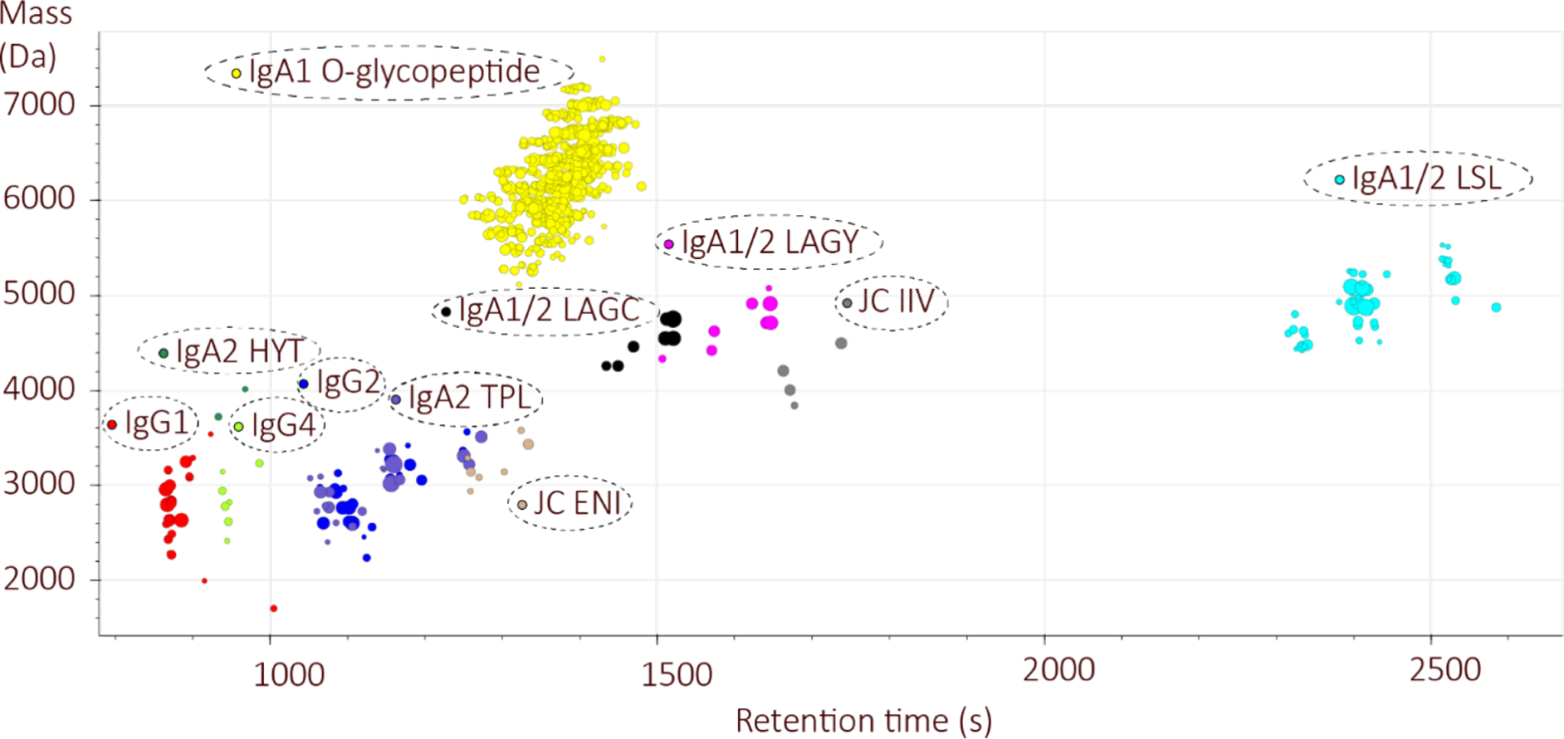
Representative IgG and IgA glycopeptide clusters detected by GlycopeptideGraphMS.

The glycopeptide identification was further extended by an open MS1 search based on mass and RT differences using GlycopeptideGraphMS [15]. RT clusters for all MS/MS assigned IgG/IgA glycopeptides were found using this tool (Figure 2). Of note, GlycopeptideGraphMS relies on the MS/MS assignment of at least one glycopeptide per RT cluster (be it automated or manual). The GlycopeptideGraphMS cluster with the highest number of connections contained the expected masses of the IgA1 *O*-glycopeptides, which were not assigned in the Byonic search (Figure S12, Supporting Information File 2). In line with the Byonic search, several other RT clusters of missed cleaved products or glycopeptides from other plasma proteins were present (data not shown).

Additional clusters with a +27.9949 Da (formylation) mass shift and an increased RT were observed for most of the IgG and IgA glycopeptides (see Figure S14, Supporting Information File 2 for representative IgG glycopeptide examples). The formylation was conveniently assigned to the glycan part (Figure S15, Supporting Information File 2) but may occur at the peptide portion as well [24–26]. Formylation is likely introduced by the exposure of the tryptic peptides to formic acid during the acid precipitation of sodium deoxycholate in the final step of the sample preparation and during subsequent storage [24]. Within the glycopeptide clusters of interest, Cys oxidation (+15.9949 Da) was assigned as an unexpected modification in all Cys-containing glycopeptides (five out of 11) at a high relative abundance (65.4–77.2%) and confirmed upon manual inspection of the MS/MS data (Figures S13 and S16–S18, Supporting Information File 2). The y- and Y-fragment ions of (glyco)peptides with Cys oxidation showed a characteristic neutral loss of 107.0041 Da (C_2_H_5_O_2_NS), as reported for singly oxidized carbamidomethylated Cys through an elimination reaction in the gas phase (Figures S13 and S16–S18, Supporting Information File 2) [27]. Peptides with Cys oxidation had a similar elution behavior as the unoxidized isomeric counterparts (with an additional hexose instead of a fucose unit), leading to a high degree of ambiguous, albeit often illogical compositions (e.g.. for the LSL cluster, Figure S16, Supporting Information File 2 and Table S2A–E, Supporting Information File 3) and false-positive assignments (e.g., the LAGC cluster, Figure S17, Supporting Information File 2) in GlycopeptideGraphMS. In line with these findings, the high number of illogical compositions and false-positive assignments of the IgA1 *O*-glycopeptide (three Cys residues) were due to modification variants on the Cys residue (Figures S12 and S13, Supporting Information File 2). In general, the assignment based on RT differences and MS1 information (manual or automated) had a highly increased uncertainty for the glycopeptides with partial Cys oxidation, and MS/MS was essential for confident identification in these cases. Of note, false-positive assignments related to Cys oxidation were also observed in the automated Byonic search upon manually reevaluation. For example, the LAGC glycopeptide composition H6N5S2 had a maximum score of 282, with no coverage of y-ions (Figure S17, Supporting Information File 2). This was due to the presence of the oxidized Cys residue at the C-terminus for which characteristic y- and Y-ions could be manually assigned in this scan. These findings substantiate that the scores in automated MS/MS searches may be still relatively high for false-positive assignments. Defining the appropriate search space with prior knowledge on relevant modifications and neutral losses is crucial to increase the identification accuracy for (glyco)peptides with unexpected modifications, such as Cys oxidation. The oxidation of Cys can appear biologically in the sample or artificially during/upon sample preparation [27–28]. In general, Met modifications are known for causing ambiguities in glycoproteomics due to partial oxidation, particularly in combination with carbamidomethylation [11,29]. To our knowledge, no study has previously reported on partial Cys oxidation as a confounder in glycoproteomics. As peptides containing the Cys oxidation had a higher abundance than the unoxidized counterparts, it is stressed that this modification should be carefully checked in Cys-containing glycopeptides as in the investigated sample, it had major implications on the IgA glycoprofiling accuracy. Further elaboration of the Cys-containing peptides, including modifications and correct glycan composition identifications, were considered beyond the scope of this study due to the largely increased complexity. Hence, the applicability of the proposed glycoproteomic data analysis workflow was demonstrated on a subset of six *N*-glycopeptide clusters, namely IgG1, IgG2/3, IgG4, JC (ENI, IIV), and IgA2 (TPL).

**Figure 3:**
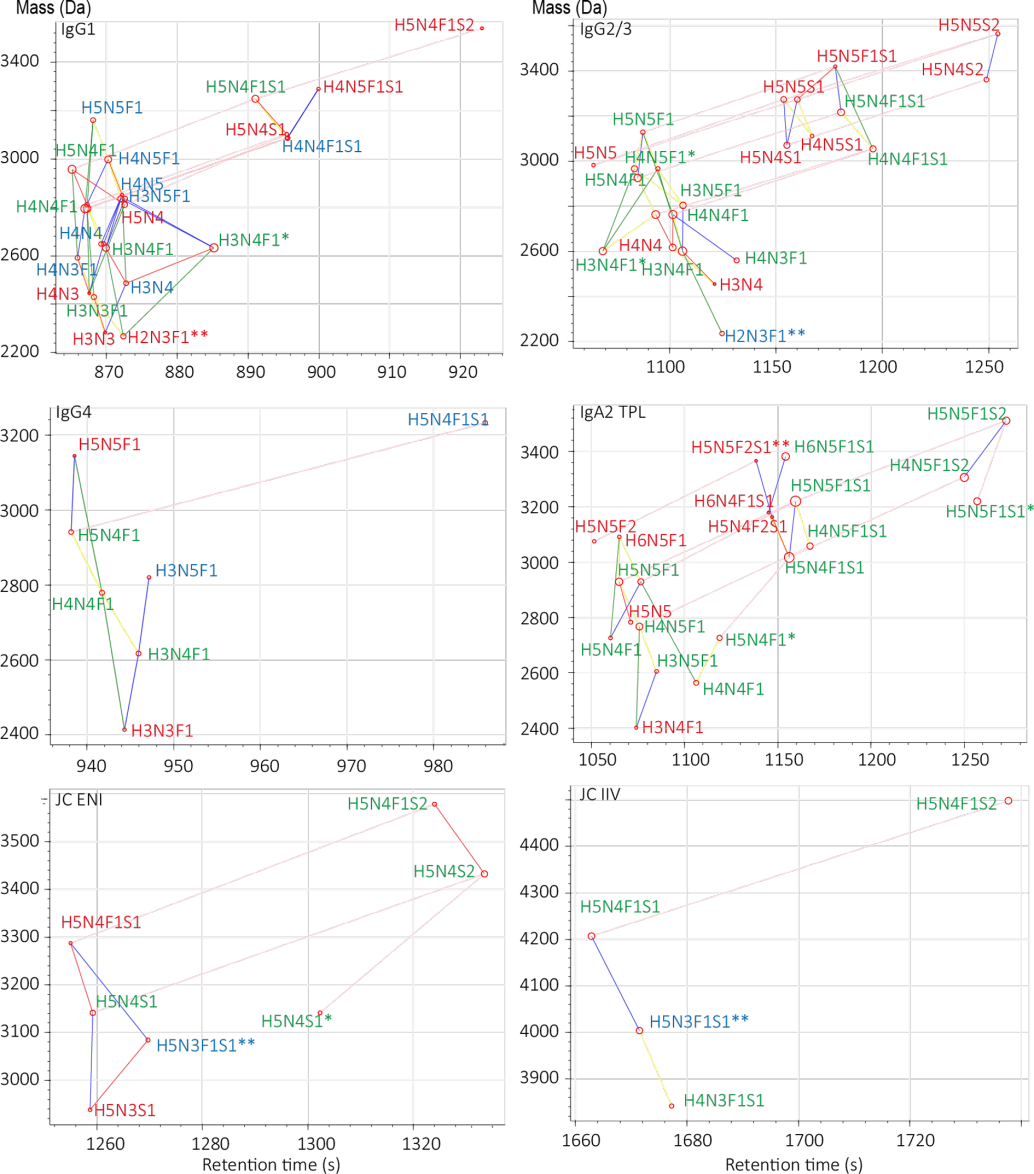
Representative GlycopeptideGraphMS output for peptides of interest. Assigned compositions were identified using MS/MS data via Byonic (green) or manual assignment (blue) or by MS1 only (red, GlycopeptideGraphMS with additional accurate-mass and isotopic pattern check of the raw data). The assignment of the compositions is based on information from all replicates. Lines between compositions indicate the mass difference for Hex (yellow), HexNAc (blue), HexHexNAc (green), Fuc (red), and NeuAc (purple). * Indicates potential deconvolution errors and ** indicates data not included in the Byonic search list.

For the six glycopeptide clusters of interest, the presence of 262 theoretical glycopeptides (based on the internal IgG/IgA glycan reference list [17] and Glyconnect entries for these peptides [20], Table S3, Supporting Information File 3) was manually evaluated in Skyline, and the presence of 83 glycopeptides in the used data was confirmed (Table S4, Supporting Information File 3). In total, 82 correct glycopeptide compositions were identified using GlycopeptideGraphMS with MS/MS validation, whereas the Byonic-only search resulted in 35 compositions (Table S4, Supporting Information File 3). Of note, four glycan compositions (H2N3F1, H2N4F1, H5N3F1S1, H5N5F2S1) were not included in the *N*-glycan search list of Byonic, and hence not included for the calculation of its glycopeptide coverage. Those glycans were only present in low abundance on the glycopeptides, and often no MS/MS spectrum was present (Figure 3). However, it highlights the importance of a complete glycan composition list for a database-based identification of glycopeptides, something that is less critical in MS1-based RT and accurate-mass-difference searches.

In the GlycopeptideGraphMS search, nine compositions were detected that were not within the internal IgG/IgA glycan reference list [17] or had an entry in Glyconnect for these peptides (Table S4, Supporting Information File 3) [20]. These analytes were present at very low relative abundances (<1%). The IgA2 TPL peptide showed the highest number (five) of additional compositions (H6N5F1, H5N5, H5N4F2S1, H5N5F2S1, and H6N4F1S1). For all glycoforms identified by GlycopeptideGraphMS, only one composition (TPL, H4N4F2S1) was determined as false-positive as no MS1 signals could be found for this analyte in the raw data. Of note, one TPL glycoform (H5N5S1) was detected with a low abundance in three out of five technical replicates but was excluded from the identification and further processing due to the presence of isobaric MS signals in the raw data. On the other hand, only one glycopeptide (IgG2/3 H3N3F1) was assigned manually, without being identified by GlycopeptideGraphMS as the correct mass and RT combination was not in the deconvoluted mass list. These false-positive and false-negative results are artifacts of the feature recognition, deconvolution, and deisotoping in OpenMS [15] prior to the GlycopeptideGraphMS analysis. Furthermore, the preprocessing steps caused some glycopeptides to be detected at multiple RT values (Figure 3), whereas the raw data showed only a single chromatographic peak. The applied OpenMS workflow has a reported accuracy of 91% for detecting the correct monoisotopic peak of a feature, and this workflow was not further optimized in the current study [15].

With respect to the consistency of the glycopeptide identification in technical replicates, the MS1-based identification (GlycopeptideGraphMS) supported by MS/MS data showed a better performance than MS/MS identification alone (47 vs 16 glycopeptides detected in all replicates, respectively, Figure S19, Supporting Information File 2). Both automated identification approaches showed variations within the data of the technical replicates, and the glycopeptide coverage was maximized by combining all measurements. For the MS1-based assignment, the variation between replicates was found in the minor glycan species, which were on the borderline of the limit of detection. Further, the stochastic nature of MS/MS selection is a known factor, which may cause variability in MS/MS-based assignments [15].

Overall, the GlycopeptideGraphMS workflow showed a high identification accuracy (82/83, 99%) and coverage (82/83, 99%, Table S4, Supporting Information File 3). In comparison, the accuracy of the Byonic search for the glycopeptides of interest was comparably high (35/37, 95%), whereas the glycopeptide coverage was moderate (35/77, 45%). This is in line with the reported near-perfect accuracy and limited coverage of the glycopeptide identification by Byonic [11]. Of note, the glycopeptide coverage of Byonic depends highly on the search parameters, fragmentation settings, and the presence and quality of MS/MS spectra. The latter is often compromised due to dynamic range limitations, especially in complex matrices [11,30]. The accuracy of both approaches (MS1 and MS/MS) may be impaired by unexpected peptide modifications, as exemplified for Cys oxidation. Thus, careful inspection of the result outputs (RT graphs in GlycopeptideGraphMS, automatically annotated MS/MS spectra in Byonic) is important. Indications of additional peptide modifications can then be considered for manual MS/MS verification and be included in the search space of automated MS/MS assignments in an iterative manner. Alternatively, a prior open search aimed at the identification of peptide modifications may be applied by software tools such as Preview [31]. Overall, this data shows that, while MS/MS-based assignment tools are essential for the confident identification of glycopeptide clusters, MS1-based approaches show a highly complementary performance by identifying glycopeptides for which no MS/MS data is present. For the latter, GlycopeptideGraphMS is a highly valuable tool as it is easy to use, fast, and open source.

### Glycopeptide curation and quantification in LaCyTools

Upon glycopeptide identification, the analytes were curated and quantified by LaCyTools. The performance of LaCyTools was compared to that of Skyline (manual curation and quantification) and GlycopeptideGraphMS (quantification). The analytes and charge states passing the quality criteria (for LaCyTools: *m*/*z* accuracy <10 ppm, isotopic pattern quality value <0.2, signal-to-noise ratio >9; for Skyline: *m*/*z* accuracy <10 ppm, idotp >0.85) were highly similar between LaCyTools and Skyline (Table S5, Supporting Information File 3). Minor differences were observed for low-abundant glycopeptides. In GlycopeptideGraphMS, quality control is only based on mass accuracy and not included in this comparison.

**Figure 4:**
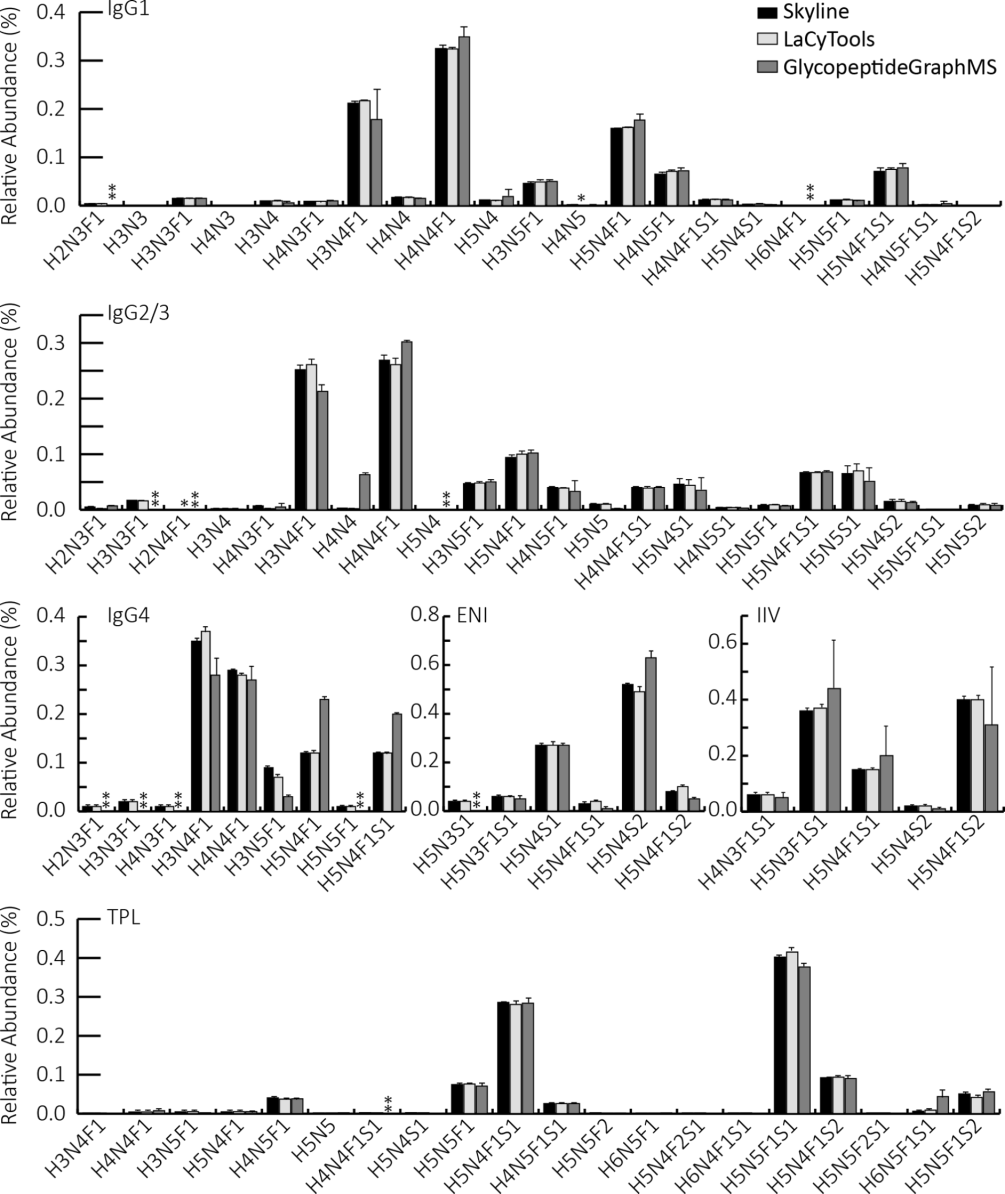
Comparison of quantification results obtained by manual integration of EICs in Skyline (black), automated integration of summed MS spectra in LaCyTools (light gray), and GlycopeptideGraphMS (dark gray). Error bars represent standard deviation of MS1-only measurements (*n* = 4 for LacyTools and Skyline; *n* = 3/4 for GlycopeptideGraphMS; in all detected replicates, *n* was at least 3. The first injection was excluded for all tools due to RT shifts and increased standard deviations). *: Did not pass the analyte curation (LaCyTools). **: Was not identified in at least 3 technical replicates (GlycopeptideGraphMS).

The three software tools evaluated for targeted glycoform quantification resulted in comparable site-specific glycosylation profiles for human plasma IgG, JC, and IgA2 (Figure 4 and Table S5, Supporting Information File 3), which were in line with the literature (Table S6, Supporting Information File 3) [17–18]. Skyline and LaCyTools showed the highest similarity in the relative quantification results (Figure S20, Supporting Information File 2). Both tools had a median relative standard deviation (RSD) of 4% over all quantified glycopeptides. In contrast, GlycopeptideGraphMS integration resulted in a higher variability (median RSD: 15%, Figure S20, Supporting Information File 2) and slightly deviating glycosylation profiles, as compared to Skyline and LacyTools. As the data used for quantification were the same, the differences in the quantification precision are caused by the data processing performed by the different software tools. Of note, the automated quantification in GlycopeptideGraphMS required additional manual interference for analytes that had multiple RTs in the output file and only a single chromatographic peak in the raw data. Similar as for the glycopeptide identification, quantification with GlycopeptideGraphMS showed clearly that the preprocessing of the data is a crucial factor for the outcome. Further optimization of the OpenMS preprocessing steps to prevent double feature assignments may improve the quantification precision.

Within the investigated quantification tools, Skyline allows the highest control of the feature selection for quantification as the integrated EICs can be manually inspected for interferences, correct peak integration, and quality criteria (mass accuracy and isotopic pattern). LaCyTools provides information on the mass accuracy and isotopic pattern and integrates the isotopes of selected features in summed MS spectra within user-defined RT windows. Here, it is crucial to select appropriate RT windows and isotopes of interest before starting the analysis to prevent the inclusion of closely eluting isomeric and isobaric interferences. Of note, isomeric glycopeptide compositions were summed and not processed individually. This approach makes RT alignment a crucial step for a robust quantification. With the optimized parameters in place, LaCyTools allows highly automated data handling, making it an excellent tool for, e.g., clinical cohort analysis. In the current work, a python script was developed to streamline the connection between GlycopeptideGraphMS identification and LaCyTools quantification (Supporting Information File 1). All tools provided absolute values for glycopeptide quantification, which were subsequently total-area-normalized per glycosylation site, as commonly done in label-free relative quantification in glycoproteomics [16–17,30,32].

In the current study, all quantitative analyses were performed on the MS1-only runs to obtain the highest possible data quality. However, runs including fragmentation scans are also suitable for quantification, albeit introducing a slightly higher variability in some cases due to a lower number of data points per chromatographic feature (in particular obvious for the IgG1 and IgG2/3 data in the current study, see Figure S21, Supporting Information File 2). The difference in the quantification accuracy between MS1-only and MS/MS data is highly dependent on the frequency of the MS1 scans, and thus the time spent on fragmentation scans. In most situations, it is likely that a compromise must be made to allow both robust quantification and data-rich MS/MS identification in the same LC–MS run. The introduction of MS1-based identification reduces the time needed for fragmentation.

## Conclusion

Here, we demonstrated a semiautomated glycoproteomics data analysis workflow for enriched glycoproteins by integrating different tools for glycopeptide identification, curation, and quantification after RPLC separation and MS(/MS) detection. For this, a mix of the human plasma-enriched antibodies IgG and IgA was used as a representative glycoproteomics sample of moderate complexity. A similar approach can be applied to a more complex sample when targeting only a select set of glycoproteins. However, to capture the full complexity of, e.g., the human glycoproteome, improvements should be made in the automated integration between the described tools. In line with previous reports on single glycoproteins, the number of identified glycoforms was significantly maximized by combining MS1-based identification (using GlycopeptideGraphMS) in combination with MS/MS-based identification (using Byonic) as compared to fragmentation-based analysis alone. Moreover, the graphical approach allowed by GlycopeptideGraphMS is very powerful for identifying unexpected glycoforms as well as modifications of the glycopeptides and aids the optimization of the search space for MS/MS annotation in an iterative manner. Although an MS1-based approach alone allows the identification of more unique glycopeptides as compared to an MS/MS-based approach, a combined workflow is essential to prevent wrongly assigned glycopeptides as well as to identify the nature of specific modifications. The combination of Byonic and GlycopeptideGraphMS identification with LaCyTools-based curation and quantification of glycopeptides from enriched glycoproteins as presented in the current work provides a powerful workflow towards high-throughput glycopeptide analysis.

## Experimental

### Sample, chemicals, and enzymes

Human plasma Visucon-F was obtained from Affinity Biologicals (Ancaster, ON, Canada). Affinity matrix beads for IgG (CaptureSelect FcXL, capacity 25–35 g/L) and IgA (CaptureSelect IgA, capacity 8 g/L) were obtained from ThermoFisher Scientific (Leiden, Netherlands). All used chemicals were from Sigma-Aldrich (Zwijndrecht, Netherlands) except for trifluoroacetic acid (Merck, Darmstadt, Germany) and acetonitrile (Biosolve, Valkenswaard, Netherlands). Purified water was used from a Purelab Ultra system (Veolia Water Technologies Netherlands B.V., Ede, Netherlands). Sequencing-grade trypsin was obtained from Promega (Madison, WI).

### Sample preparation

A detailed description of the methods for the immunoaffinity enrichment of the immunoglobulins and the glycopeptide preparation can be found elsewhere [17]. In brief, 5 µL of Visucon F plasma standard were diluted in PBS, and the immunoglobulins were enriched using a mix of CaptureSelect FcXL Affinity matrix beads for IgG and CaptureSelect IgA affinity matrix beads for IgA. Upon incubating the serum and the beads for 1 h at room temperature with agitation, the beads were washed three times with PBS and three times with water. The immunoglobulins were released by acid elution (100 mM formic acid) and collected into a 96-well PCR plate (Greiner Bio-One, Kremsmünster, Austria). Finally, the eluates were dried for 2.5 h at 60 °C by centrifugation under vacuum.

For tryptic digestion, the dried sample was reconstituted in 10 µL of reduction–alkylation buffer containing 100 mM Tris buffer, 1% w/v SDC, 10 mM tris(2-carboxyethyl)phosphine (TCEP), and 40 mM chloroacetamide (CAA). Upon mixing for 5 min, the samples were incubated for 5 min at 95 °C and cooled to room temperature. Tryptic digestion was started by the addition of 50 µL digestion buffer containing 50 mM ammonium bicarbonate pH 8.5 and 200 ng sequencing-grade trypsin. Upon mixing for 5 min, the sample was incubated at 37 °C overnight. Acid precipitation using 1.2 µL formic acid was performed on the following day. The precipitate was removed by centrifugation, and 40 µL of the supernatant was transferred to a V-bottom 96-well plate (Greiner). The sample was stored at −20 °C.

### LC–MS/MS analysis

A 0.5 µL aliquot of the sample was analyzed five times with MS1 only (for MS1-based identification in GlycopeptideGraphMS and quantification in LaCyTools, Skyline, and GlycopeptideGraphMS) and five times with additional MS/MS (for fragmentation-based identification using Byonic and quantification using LaCyTools) in an alternating order. For the separation of the (glyco)peptides, the sample was injected into an Easy nLC 1200 system (Thermo Fisher Scientific) equipped with an in-house prepared precolumn (15 mm × 100 μm; Reprosil-Pur C18-AQ 3 μm, Dr. Maisch, Ammerbuch, Germany) and an analytical nanoLC column (15 cm × 75 μm; Reprosil-Pur C18-AQ 3 μm). As mobile phases 0.1% formic acid in water (A) and 20% water/80% acetonitrile + 0.1% formic acid (B) were used. A gradient from 10–40% of the mobile phase B was applied within 20 min. The LC was hyphenated to an Orbitrap Fusion Lumos MS (Thermo Fisher Scientific). For MS1 analysis, scans were acquired in a mass range of *m*/*z* 400–3,500 in positive mode. The resolution was set to 120,000. The target for automatic gain control (AGC) was set to 400,000. The maximum injection time was 50 ms. An intensity threshold of 20,000 was applied. For MS/MS analysis, charge states 2–7 were included for stepped higher-energy C-trap dissociation (HCD) with a normalized collision energy (NCE) of 35% ± 5% (30%, 35%, and 40% combined in one spectrum), a maximum injection time of 60 ms, and a AGC target of 50,000. Additionally, MS/MS fragmentation was triggered for a HexNAc loss (204.087). For the triggered MS/MS analysis, a stepped HCD with an NCE of 35% ± 15% (20%, 35%, and 50% combined in one spectrum) was applied, and the AGC target was increased to 500,000 while the maximum injection time was increased to 200 ms. For all MS/MS scans, a precursor isolation width of *m*/*z* 1.2 was used. The MS/MS scan resolution was 30,000 and the *m*/*z* range was 110–3,500.

### MS/MS data evaluation

A manual inspection of the raw data was performed in Xcalibur (v. 2.2, Thermo Fisher Scientific). PMI-Byonic (v. 3.7.13 Protein Metrics) was used for the MS/MS-based protein and glycosylation site identifications [14]. Protein identification was based on a canonical *Homo sapiens* UniProt database including 71,591 protein sequences (20,205 from Swiss-Prot and 51,386 from TrEMBL). The C-terminal cleavage of lysine and arginine and a maximum of two missed cleavages was allowed. A tolerance of 10 ppm was applied for the precursors and 20 ppm for fragment ions. A carbamidomethylation was set as a fixed modification for cysteine residues. Methionine oxidation was enabled as a variable modification. The search for *N*- and *O*-glycopeptides was separately performed. For this purpose, either the database “*N*-glycan 309 mammalian no sodium” (Supporting Information File 4) or “*O*-glycan 78 mammalian” (Supporting Information File 5) was applied as a custom modification. For manual MS/MS assignments, the web tool ProteinProspector v. 6.2.1 was used (http://prospector.ucsf.edu/prospector/cgi-bin/msform.cgi?form=msproduct). All glycopeptide compositions that were not identified by Byonic were subjected to a manual check of the MS/MS raw data in Xcalibur. This check included verifying the presence of the characteristic MS/MS ions (Table S4, Supporting Information File 3). In addition, allotypes of IgG3 and IgA2, which can be present in a human plasma pool [3], were manually checked. For this, the peptide sequences TKPWEEQYNSTFR, GFYPSDIAVEWESSGQPENNYNTTPPMLDSDGSFFLYSK (IgG3 *N*-glycopeptides), and MAGKPTHINVSVVMAEADGTC(Y) (IgA2 *N*-glycopeptide) were checked for the presence of the Y1 (peptide + HexNAc) ion in the MS/MS data. In addition, the expected glycoforms H1N1, H1N1S1, and H1N1S2 of the IgG3 *O*-glycopeptide SCDTPPPCPR were checked.

### GlycopeptideGraphMS analysis

MS1-based glycopeptide identification in all five MS1-only measurements and visualization was performed using GlycopeptideGraphMS (v. 2.06) according to the user manual [15]. In short, the raw data were first transformed to the mzML format using msconvert (ProteoWizard 3.0 suite). The data preprocessing included the deconvolution of all MS1 signals using an OpenMS workflow (KNIME_OPENMS_GraphMS_Preprocessing_120318) in KNIME [15,33–34]. This workflow was used with OpenMS 2.3. Adaptions in the parameters were made in the *m*/*z* range of 400–3500 and the charge states 2–7. For the glycopeptide identification in GlycopeptideGraphMS, the intensity threshold was set to 1,000,000, the allowed mass deviation of the glycan building blocks to 0.02 Da, and the maximum subgroup degree was set to 1. As composition searching blocks (see the example provided in Supporting Information File 6), hexose (Hex, 162.0528 Da, max. 30 s RT difference). *N-*Acetylhexosamine (HexNAc, 203.0794 Da, max. 30 s RT difference), hexose, and *N*-acetylhexosamine (HexHexNAc, 365.1322 Da, max. 30 s RT difference), deoxyhexose (Fuc, 146.0579 Da, max. 20 s RT difference), and *N*-acetylneuraminic acid (NeuAc, 291.0954 Da, max. 120 s RT difference) were enabled. For each glycopeptide cluster of interest, one data point was assigned to a composition that was verified by the Byonic search. For the visualization in GlycopeptideGraphMS, the diameter of the data points and the relative abundance of the glycopeptides were represented upon logarithmic scaling between intensities from 1 × 10^6^ to 1 × 10^12^. False-positive assignments containing negative values in the compositions (illogical compositions) based on the assigned reference data points of all glycopeptides were removed. Analytes (with logical compositions) connected solely to analytes with illogical compositions (i.e., negative features) were excluded as well. For quantitative comparisons, only analytes were considered which were identified in at least three technical replicates. Intensities of analytes present at more than one RT were summed in case of a close RT proximity (likely isomers) or manually checked in the raw data for multiple peaks and included or excluded, dependent on the presence of multiple peaks in the raw data.

### Skyline analysis

In addition to the automated glycopeptide identification, a MS1 assignment and peak integration was performed in Skyline (v19.1.0.193). The correct peak integration was manually checked. A reference glycopeptide composition list was inserted into Skyline. This list contained the merged information from the automatically assigned compositions (Byonic and GlycopeptideGraphMS), compositions listed on GlyConnect [20] for IgG and IgA, and an in-house analyte list that was recently used for an IgG/IgA analysis (based on literature information and manual peak assignment in MS1) [17]. The transition settings were set to product ions, the charge states were set to 2–7, and the time window was adjusted for each different glycopeptide cluster. MS1 data of the glycopeptide compositions were manually inspected, and charge states with an isotope dot product (idotp) >0.85 and a mass accuracy <10 ppm were included. “Normalized Area” was used for quantification.

### LaCyTools analysis

For automated quantification in LaCyTools (v 1.0.1) [16], the raw data were converted to the mzXML format by MSConvert. The generation of the LaCyTools analyte list was supported by an in-house Python (v 3.7.6) script (Supporting Information File 1), which converted a representative GlycopeptideGraphMS output to the required input format for LaCyTools. Glycopeptide compositions that were not assigned in the representative data set in GlycopeptideGraphMS were added to the list to an appropriate retention time cluster. Potentially false-positive results (no MS1 isotope pattern matching or no MS/MS verification) were manually removed. The applied analyte list is provided in Supporting Information File 6. Next, an alignment list was created by selecting the most abundant glycopeptide compositions for each RT cluster. The width of the retention time cluster was set to 15 s and adjusted to 7 s for analytes with closely eluting interference signals. The RT alignment of the technical replicates was performed within a time window of 30 s and an *m*/*z* window of 0.1. For analyte curation and quantification, an *m*/*z* window of 0.025 was used. Upon processing in LaCyTools, all charge states of analytes with an isotopic pattern quality value higher than 0.2, mass accuracies of >10 ppm, and a signal-to-noise ratio <9 were excluded. The peak areas of the remaining charge states were summed and corrected by being divided by the isotopic pattern fraction. Of note, for the comparison of the relative quantification of GlycopeptideGraphMS, Skyline, and LaCyTools, the relative abundance was not renormalized to the intersection of the analytes.

## Supporting Information

Raw data were made available in MassIVE: https://doi.org/doi:10.25345/C5JJ00.

File 1 Supporting tables.

File 2 Supporting figures.

File 3 Python script connecting the GlycopeptideGraphMS output and LaCyTools input.

File 4 LaCyTools analyte list.

File 5 Analyte search list.

File 6 Byonic *N*-glycan list.

File 7 Byonic *O*-glycan list.
