# Supplementary material for: Semiautomated glycoproteomics data analysis workflow for maximized glycopeptide identification and reliable quantification
Source: Beilstein J Org Chem. 2020 Dec 11;16:3038–51. doi: 10.3762/bjoc.16.253 (PMC7736696; doi:10.3762/bjoc.16.253)
Supplement: File 2 — Supporting figures. [file Beilstein_J_Org_Chem-16-3038-s002.pdf]

BEILSTEIN JOURNAL OF ORGANIC CHEMISTRY

Supporting Information

for

Semiautomated glycoproteomics data analysis workflow for maximized glycopeptide identification and reliable quantification

Steffen Lippold, Arnoud H. de Ru, Jan Nouta, Peter A. van Veelen, Magnus Palmblad, Manfred Wuhrer and Noortje de Haan

Beilstein J. Org. Chem. **2020,** *16,* 3038–3051. doi:10.3762/bjoc.16.253

Supporting figures

License and Terms: This is a supporting information file under the terms of the Creative Commons Attribution License (https://creativecommons.org/licenses/by/4.0). Please note that the reuse, redistribution and reproduction in particular requires that the author(s) and source are credited and that individual graphics may be subject to special legal provisions.
The license is subject to the *Beilstein Journal of Organic Chemistry* terms and conditions: (https://www.beilstein-journals.org/bjoc/terms)

Contents

Figure S1: Byonic MS/MS assignment of IgG1 glycopeptide (H5N4F1). 3
Figure S2: Byonic MS/MS assignment of IgG2/3 glycopeptide (H3N4F1). 3
Figure S3: Byonic MS/MS assignment of IgG4 glycopeptide (H3N4F1). 4
Figure S4: Byonic MS/MS assignment of LSL glycopeptide (H5N4S1). 4
Figure S5: Byonic MS/MS assignment of LAGY glycopeptide (H5N5F1S2). 5
Figure S6: Byonic MS/MS assignment of LAGC glycopeptide (H5N5F1S2). 5
Figure S7: Byonic MS/MS assignment of TPL glycopeptide (H5N4F1S1). 6
Figure S8: Byonic MS/MS assignment of HYT glycopeptide (H5N5F1S1)...................... 6
Figure S9: Byonic MS/MS assignment of ENI glycopeptide (H5N4S2). 7
Figure S10: Byonic MS/MS assignment of IIV glycopeptide (H5N4F1S1). 7
Figure S11: Byonic MS/MS assignment of an additional isobaric IgG3 glycopeptide (H3N4F1). 8
Figure S12: IgA1 *O*-glycopeptide cluster analysis by GlycopeptideGraphMS. 9
Figure S13: Representative manual MS/MS assignments of IgA1 *O*-glycopeptides. 10
Figure S14: Representative RT clusters of formylated IgG glycopeptides. 11
Figure S15: MS/MS assignment of glycan formylation. 11
Figure S16: IgA1/2 LSL glycopeptide cluster.. 12
Figure S17: IgA1/2 LAGC glycopeptide cluster. 13
Figure S18: IgA2 HYT glycopeptide cluster.. 14
Figure S19: Evaluation of MS1 based and MS2 based glycopeptide identification consistency. 15
Figure S20: Similarity assessment of different quantification tools. 16
Figure S21: Relative quantification results of MS1 vs. MS2 in LaCyTools. 17

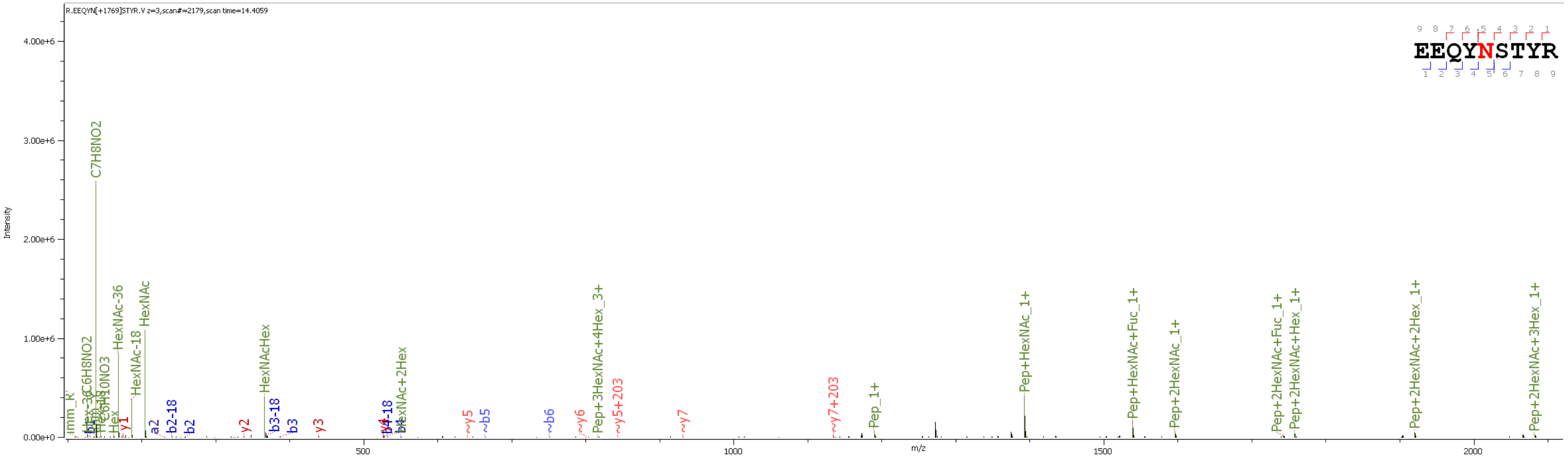

Figure S1: Byonic MS/MS assignment of IgG1 glycopeptide (H5N4F1).

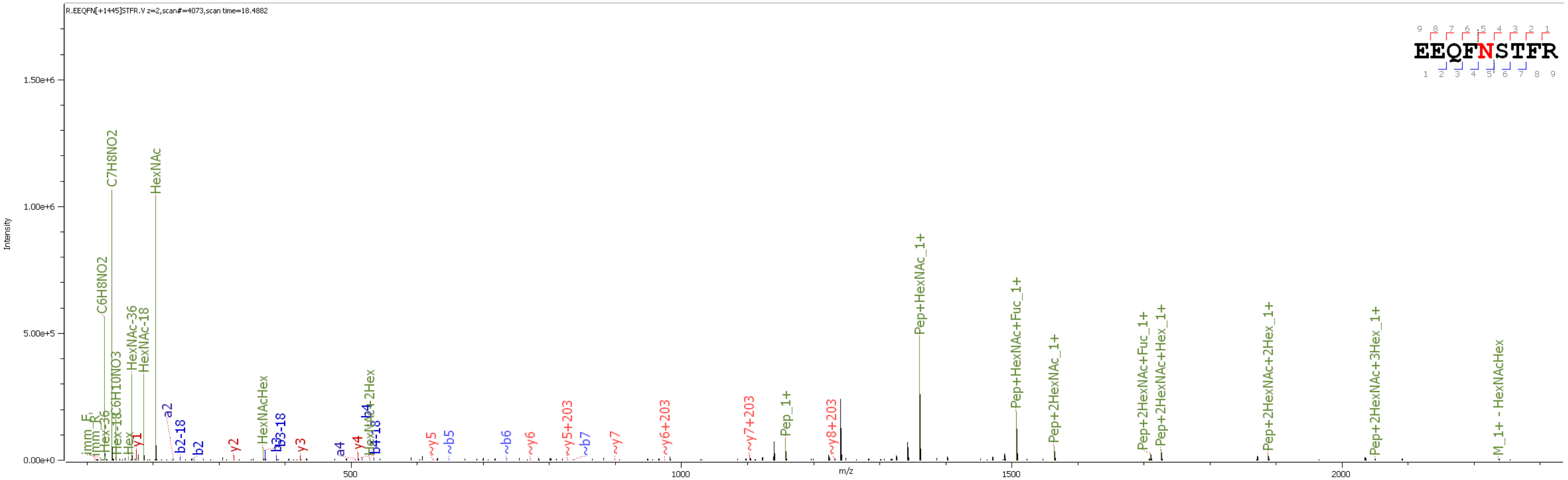

Figure S2: Byonic MS/MS assignment of IgG2/3 glycopeptide (H3N4F1).

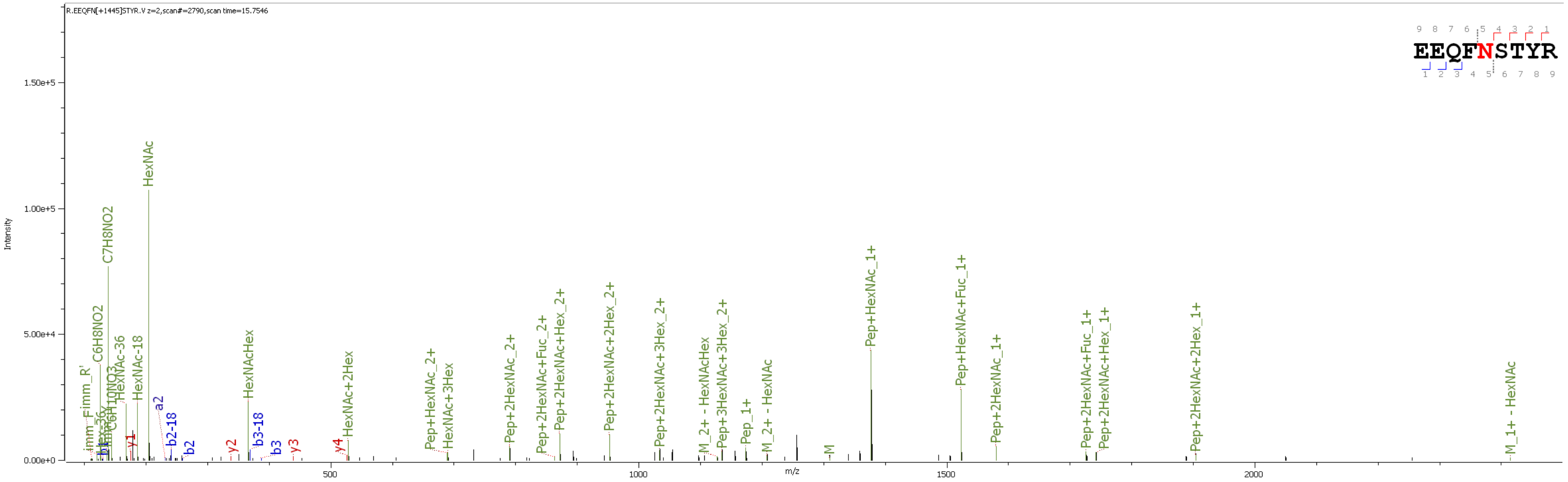

Figure S3: Byonic MS/MS assignment of IgG4 glycopeptide (H3N4F1).

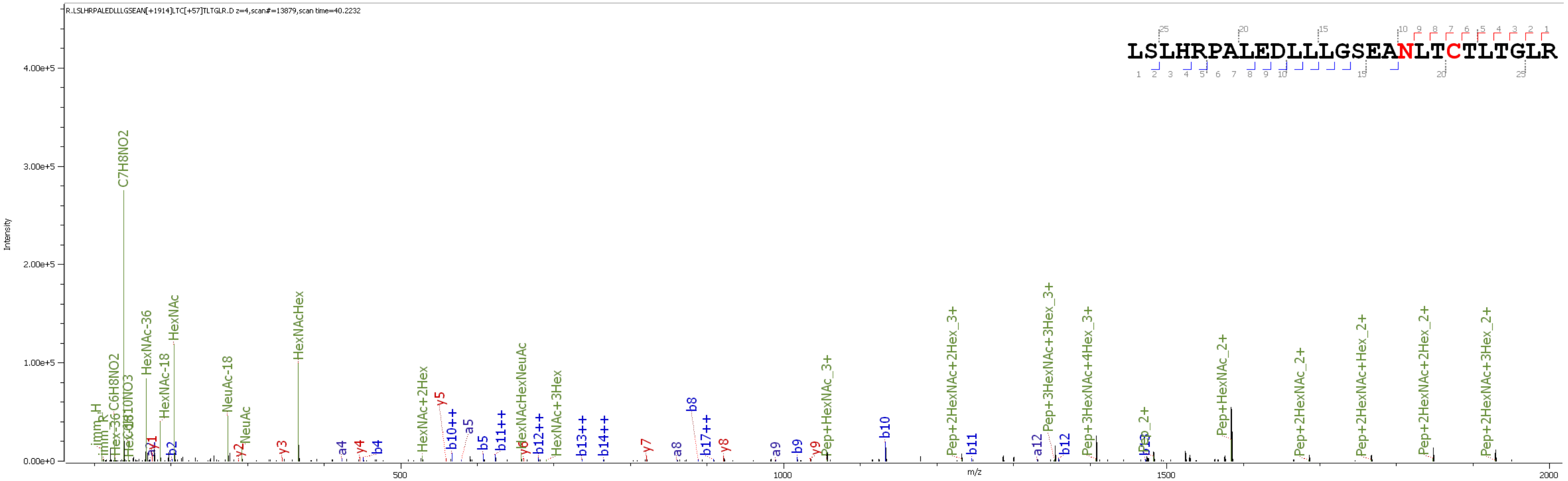

Figure S4: Byonic MS/MS assignment of LSL glycopeptide (H5N4S1).

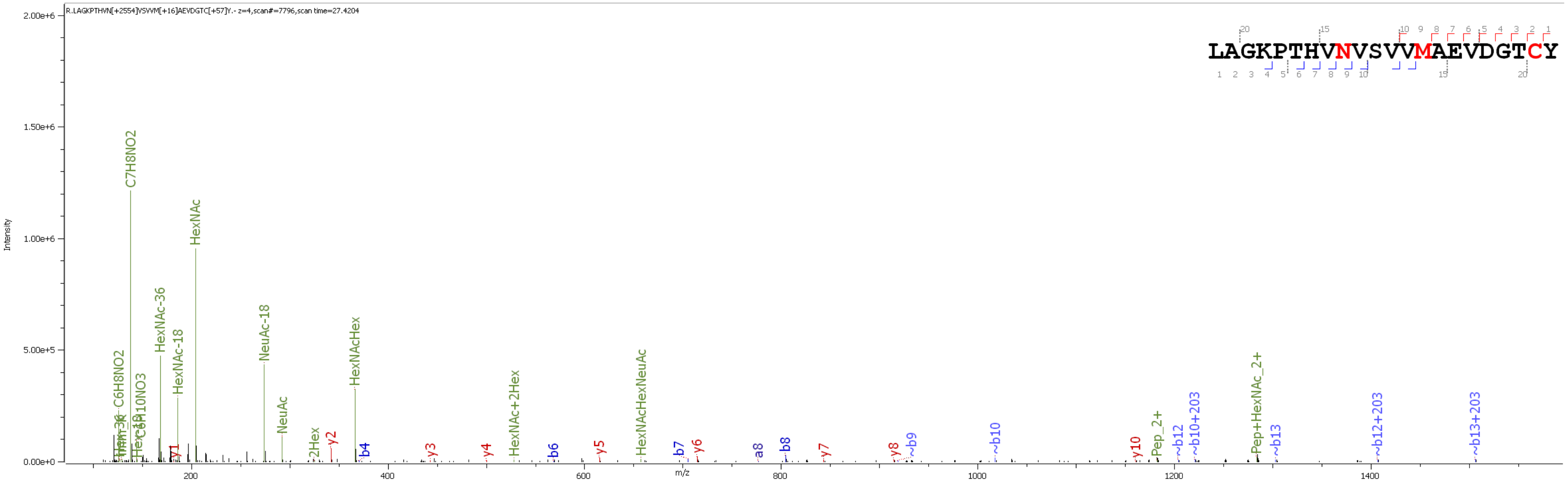

Figure S5: Byonic MS/MS assignment of LAGY glycopeptide (H5N5F1S2).

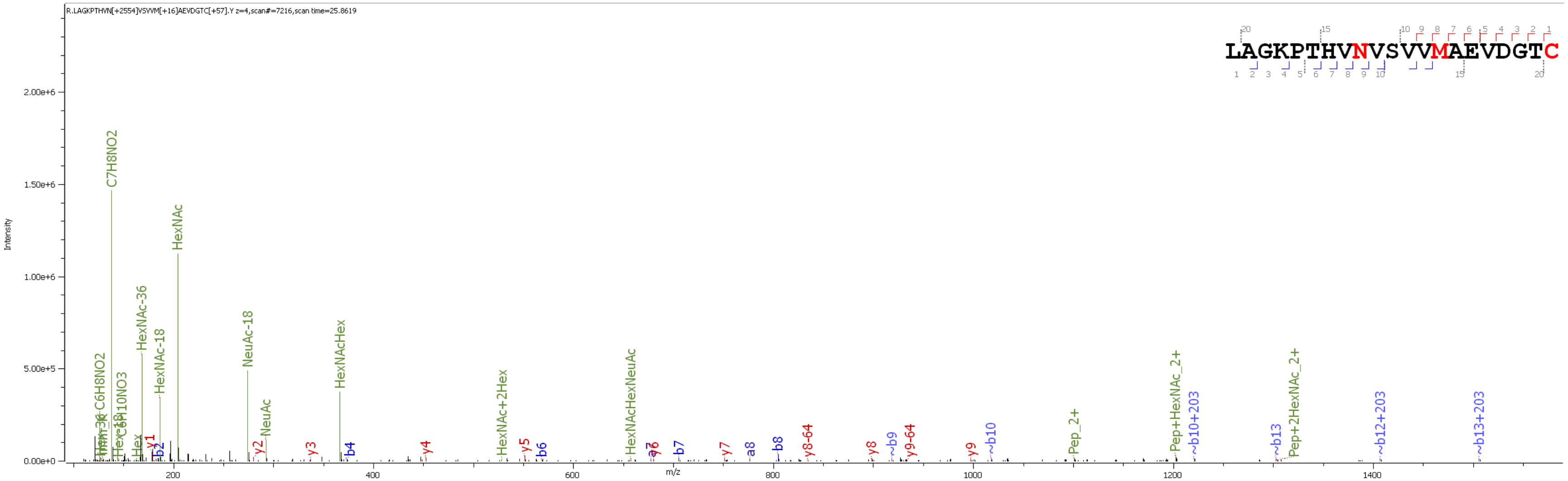

Figure S6: Byonic MS/MS assignment of LAGC glycopeptide (H5N5F1S2).

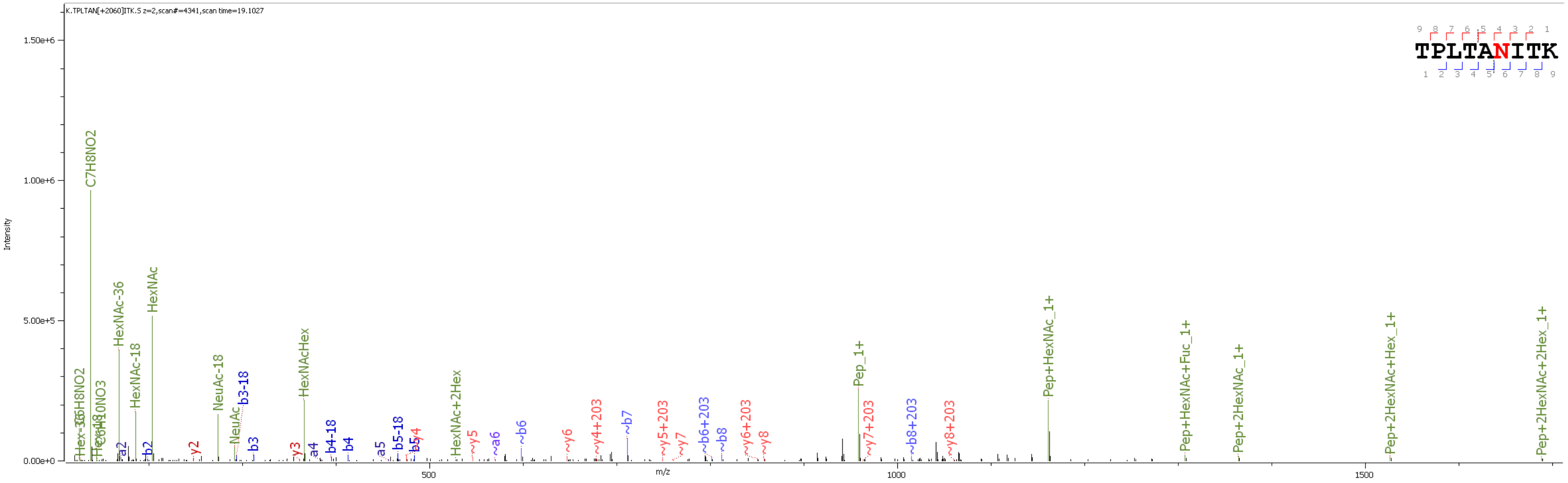

Figure S7: Byonic MS/MS assignment of TPL glycopeptide (H5N4F1S1).

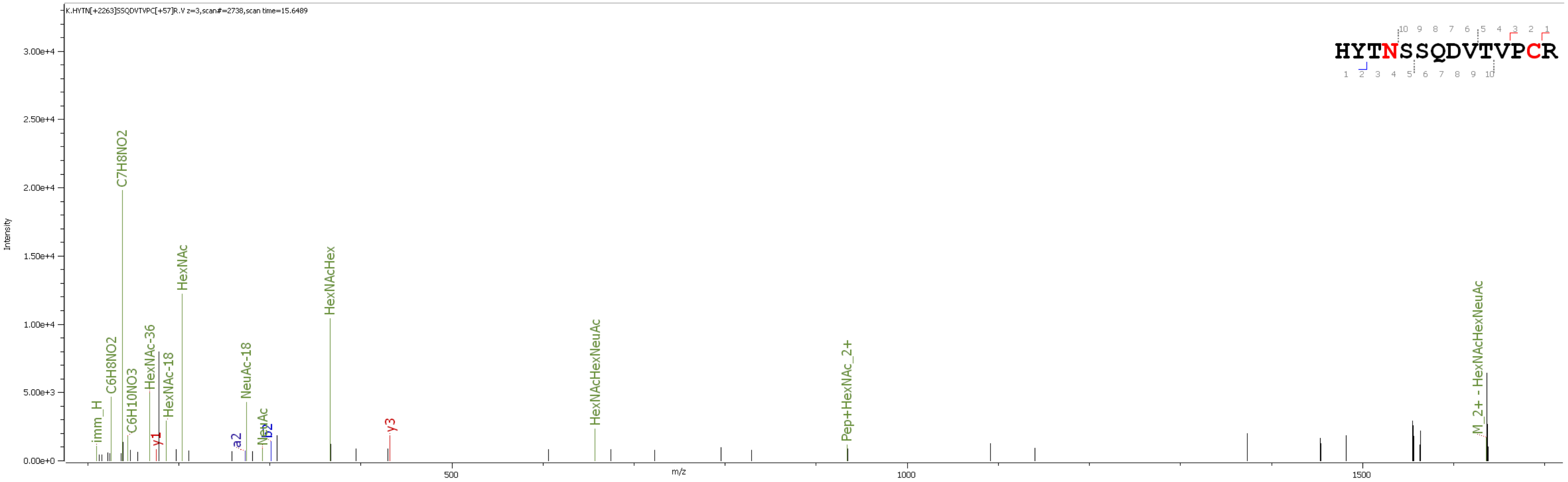

Figure S8: Byonic MS/MS assignment of HYT glycopeptide (H5N5F1S1).

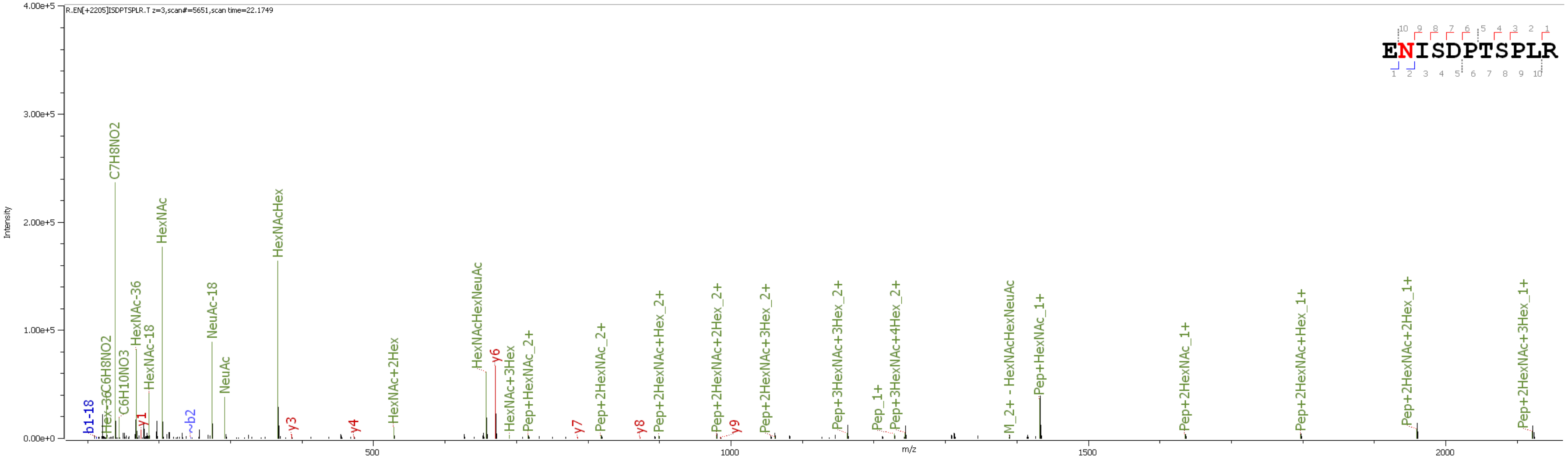

Figure S9: Byonic MS/MS assignment of ENI glycopeptide (H5N4S2).

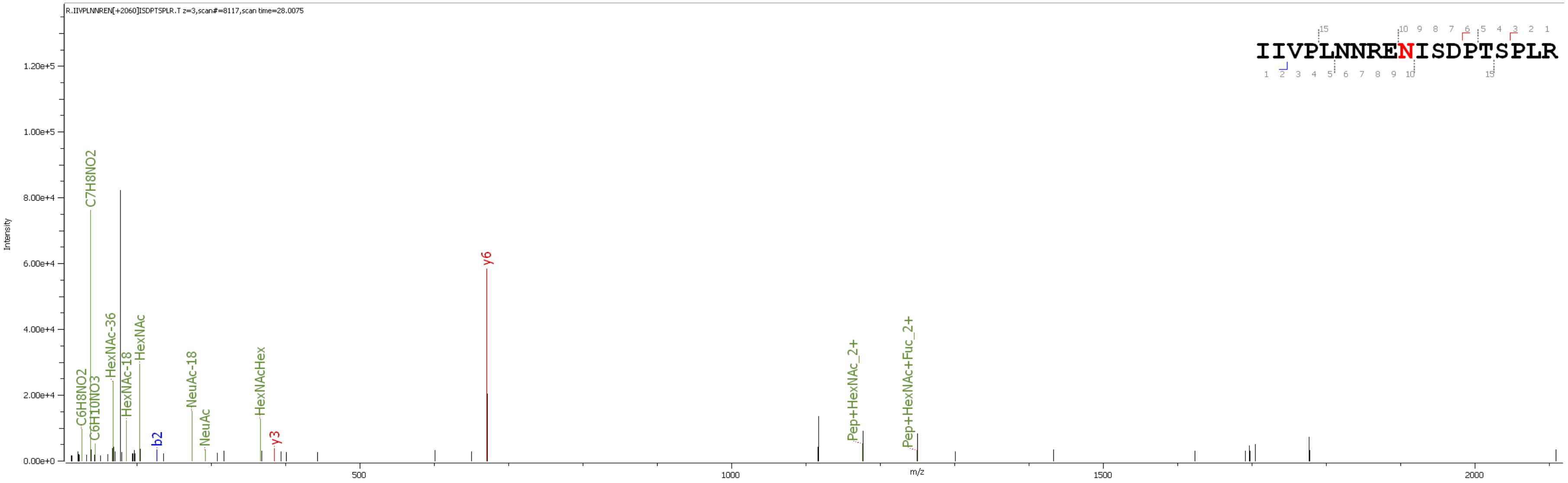

Figure S10: Byonic MS/MS assignment of IIV glycopeptide (H5N4F1S1).

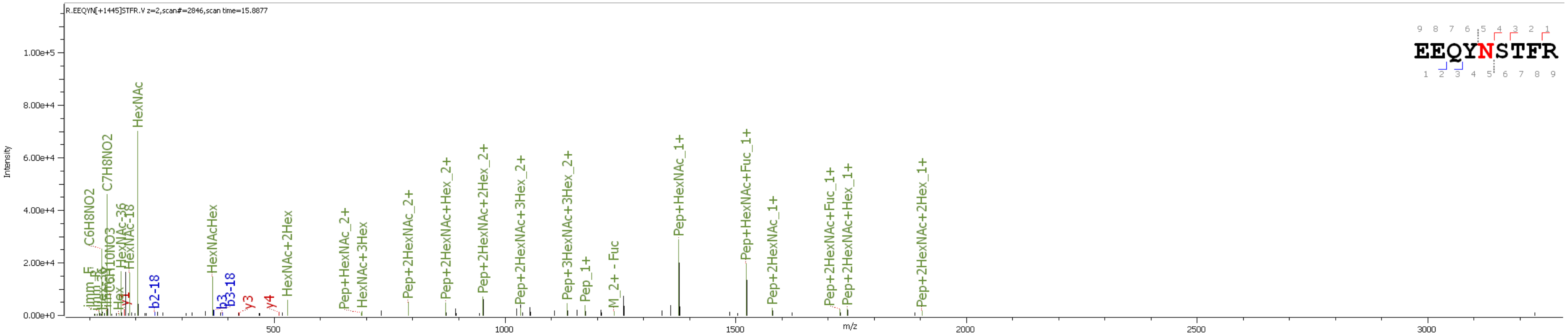

Figure S11: Byonic MS/MS assignment of an additional isobaric IgG3 glycopeptide (H3N4F1).

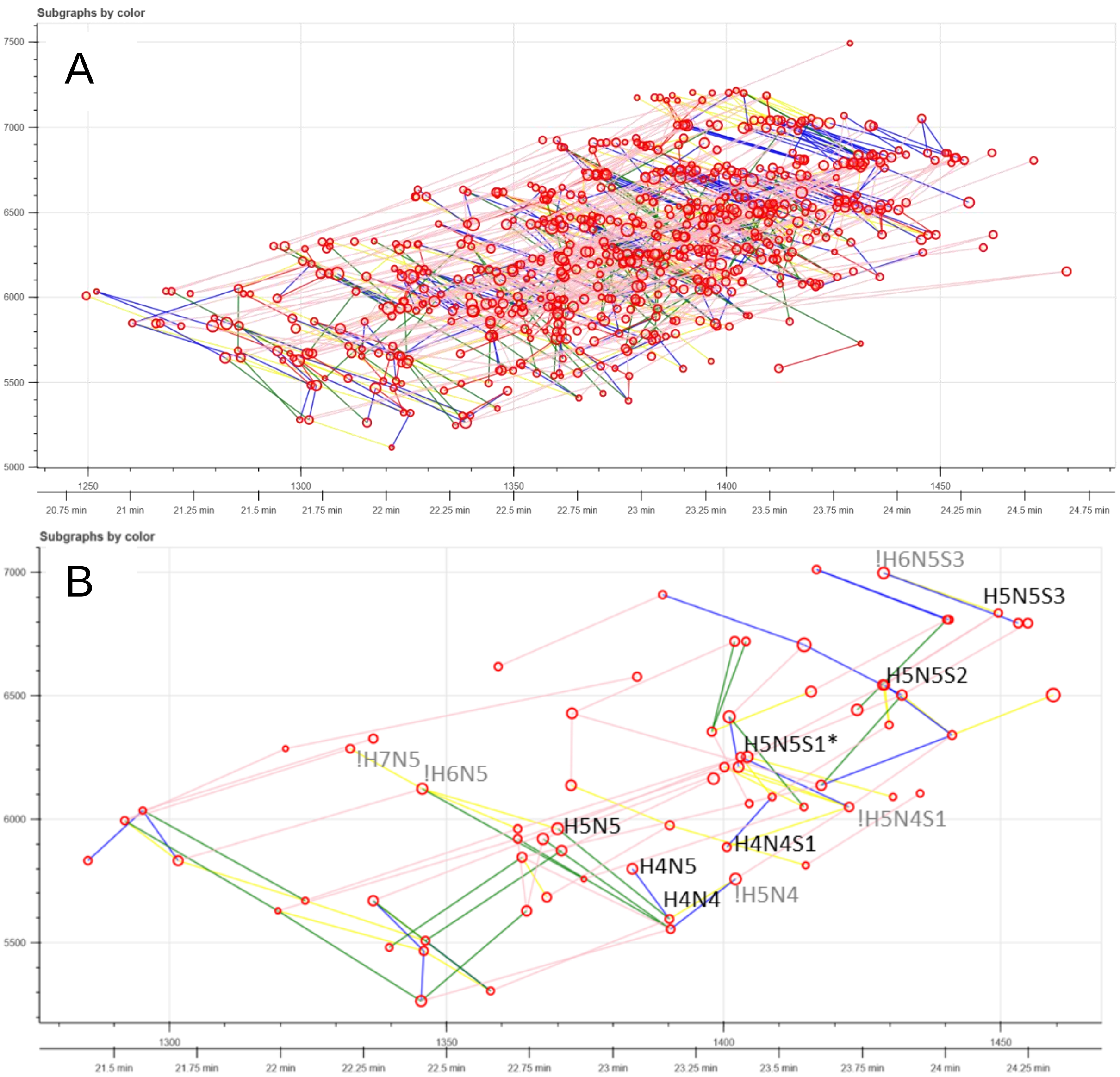

Figure S12: IgA1 *O*-glycopeptide cluster analysis by GlycopeptideGraphMS. Peptide sequence: HYTNPSQDVTVPCPVPSTPPTPSPSTPPTPSPSCCHPR (A) With fucose as searching block and (B) without fucose as searching block. Reported IgA1 *O*-glycopeptide compositions [1] are in part annotated (black, *assigned manually by MS/MS, **Figure S13**). Mass differences of fucose building blocks were detected (A), which resulted actually from Cys oxidation (**Figure S13**) and a mass difference of a hexose. As fucose is not an expected glycan building block of IgA1 *O*-glycans [2], a GlycopeptideGraphMS analysis was performed without fucose (B). Several compositions contained a higher number of Hex residues than HexNAc residues (annotated in gray in (B)). This is also not expected for IgA1 *O*-glycans [2] and results from a combination of Cys oxidation and incomplete carbamidomethylation. The mass difference of an oxidized and a carbamidomethylated glycopeptide has the same mass difference as Hex and HexNAc leading to ambiguities as reported previously [3]. Further investigation of the peptide modifications was hampered by the amount and the low information content of MS/MS spectra. Line colors: Yellow (Hex), Blue (HexNAc), Green (HexNAcHex), Red (Fucose), Pink (NeuAc).

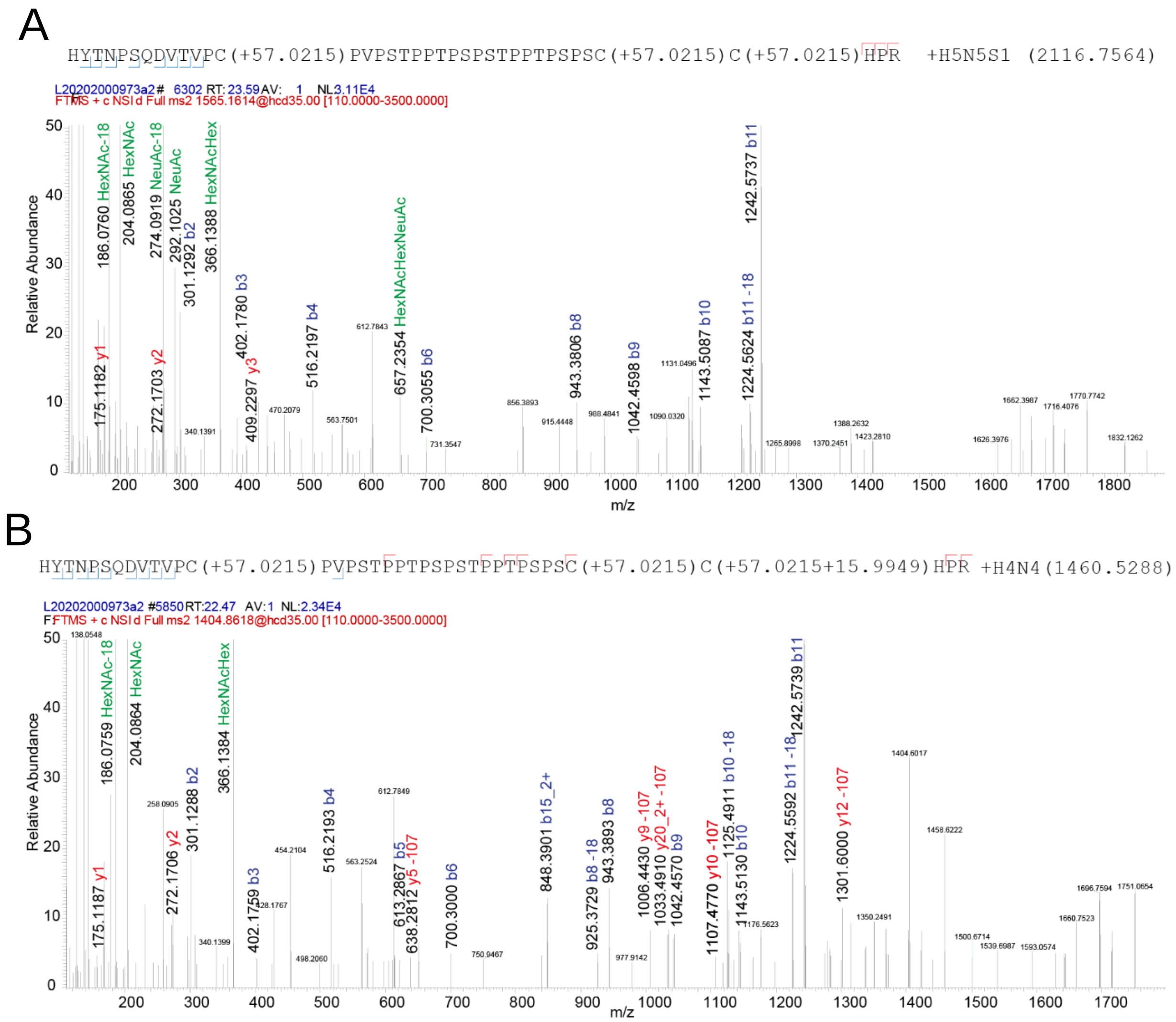

Figure S13: Representative manual MS/MS assignments of IgA1 *O*-glycopeptides. Glycopeptides were fragmented carrying the total glycan composition (A) H5N5S1 and (B) H4N4 with 1 out of 3 cysteines oxidized and carbamidomethylated. The position of the additional oxidation is at one of the two C-terminal cysteines but the exact position could not be determined.

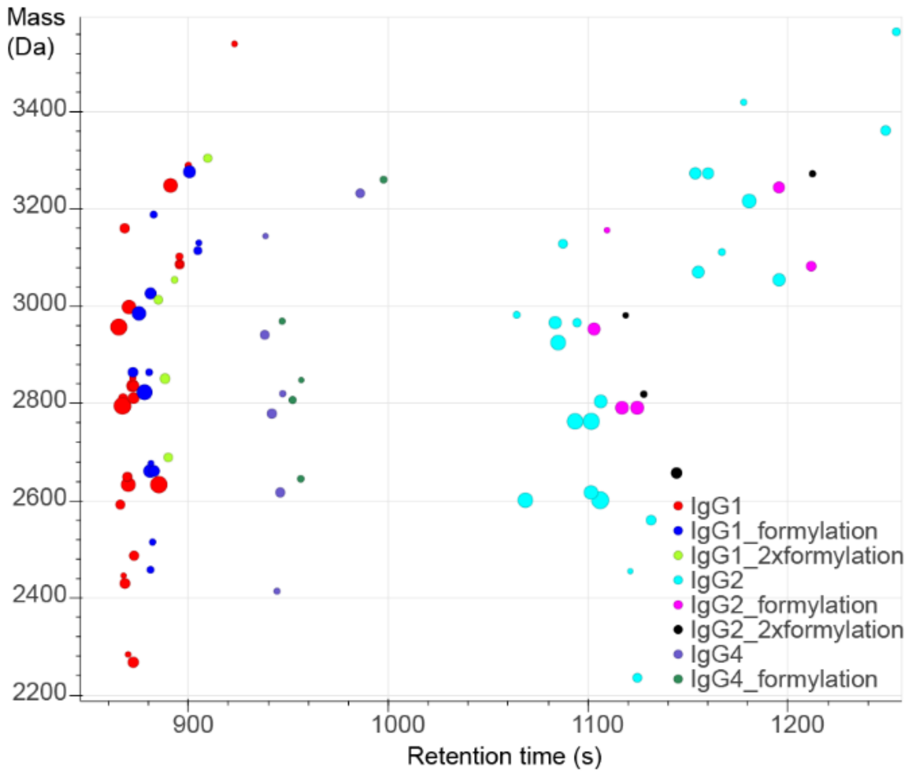

Figure S14: Representative RT clusters of formylated IgG glycopeptides. All other glycopeptides showed formylation as well (data not shown).

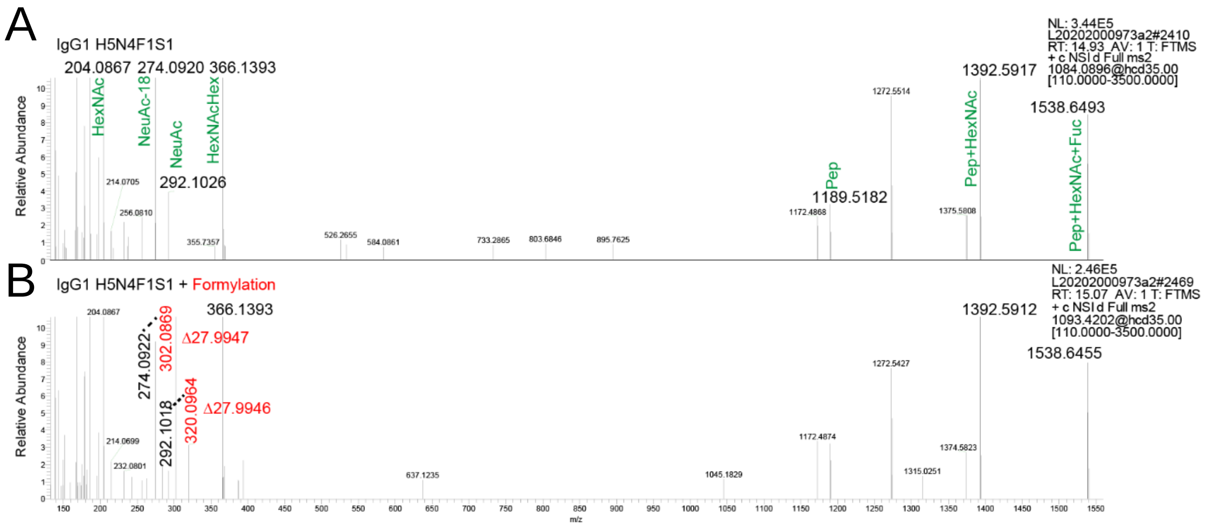

Figure S15: MS/MS assignment of glycan formylation. Representative MS/MS assignment of (A) unmodified and (B) formylated sialic acid of the IgG1 H5N4F1S1 glycopeptide.

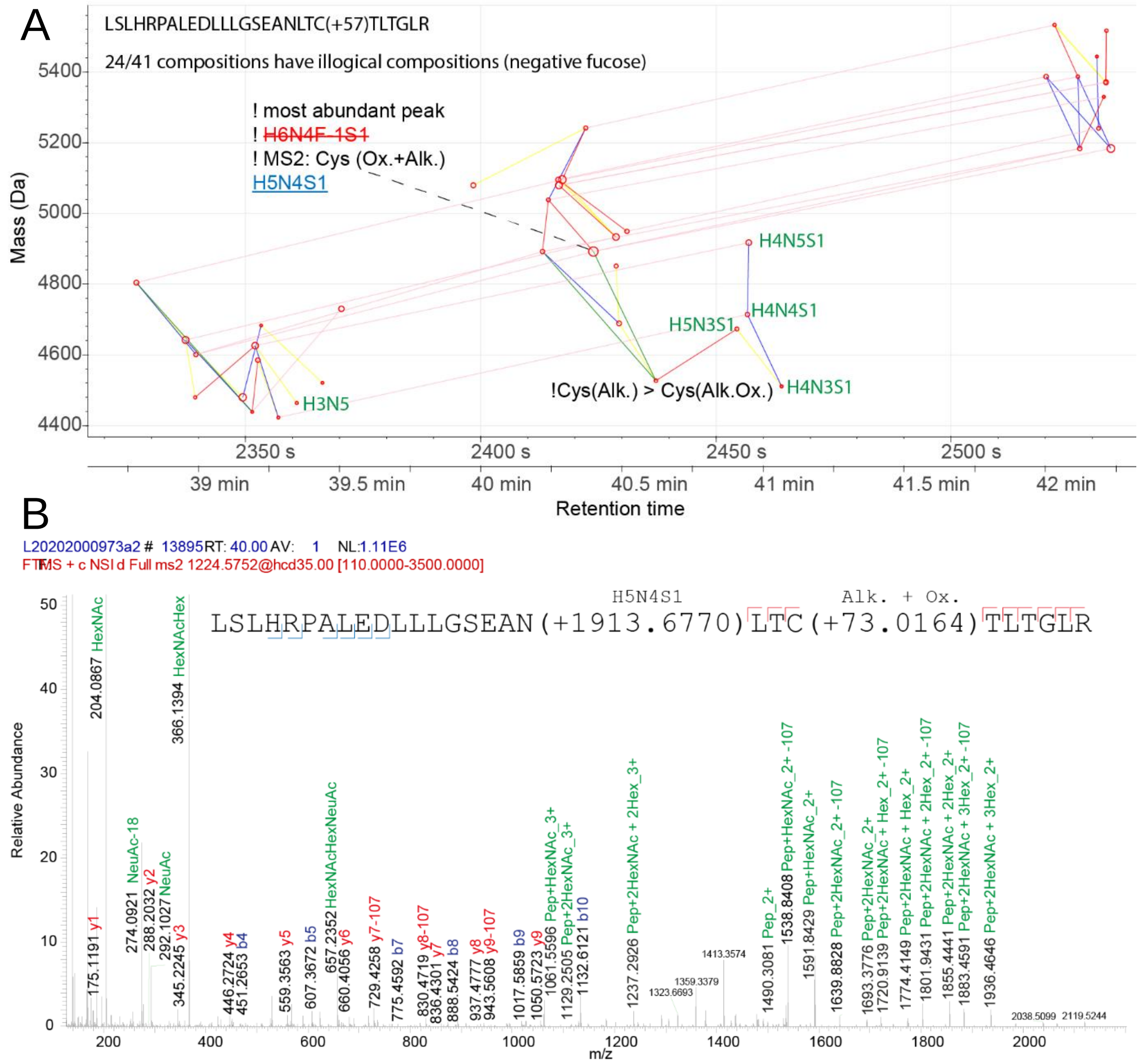

Figure S16: IgA1/2 LSL glycopeptide cluster. (A) Representative GlycopeptideGraphMS analysis of IgA1/2 LSL glycopeptide. Compositions in green were assigned in Byonic. (B) Manual MS/MS assignment of H5N4S1 glycoform with Cys oxidation*. Line colors (A): Yellow (Hex), Blue (HexNAc), Green (HexNAcHex), Red (Fucose), Pink (NeuAc).

*The Cys oxidation rate was calculated representatively for H5N4S1 in Skyline (65.4% ± 11.8%) as fractions of the oxidized product of the overall intensities from the peptide with oxidized Cys and the expected cysteine containing peptides based on peak areas of extracted ion chromatograms in Skyline (MS1).

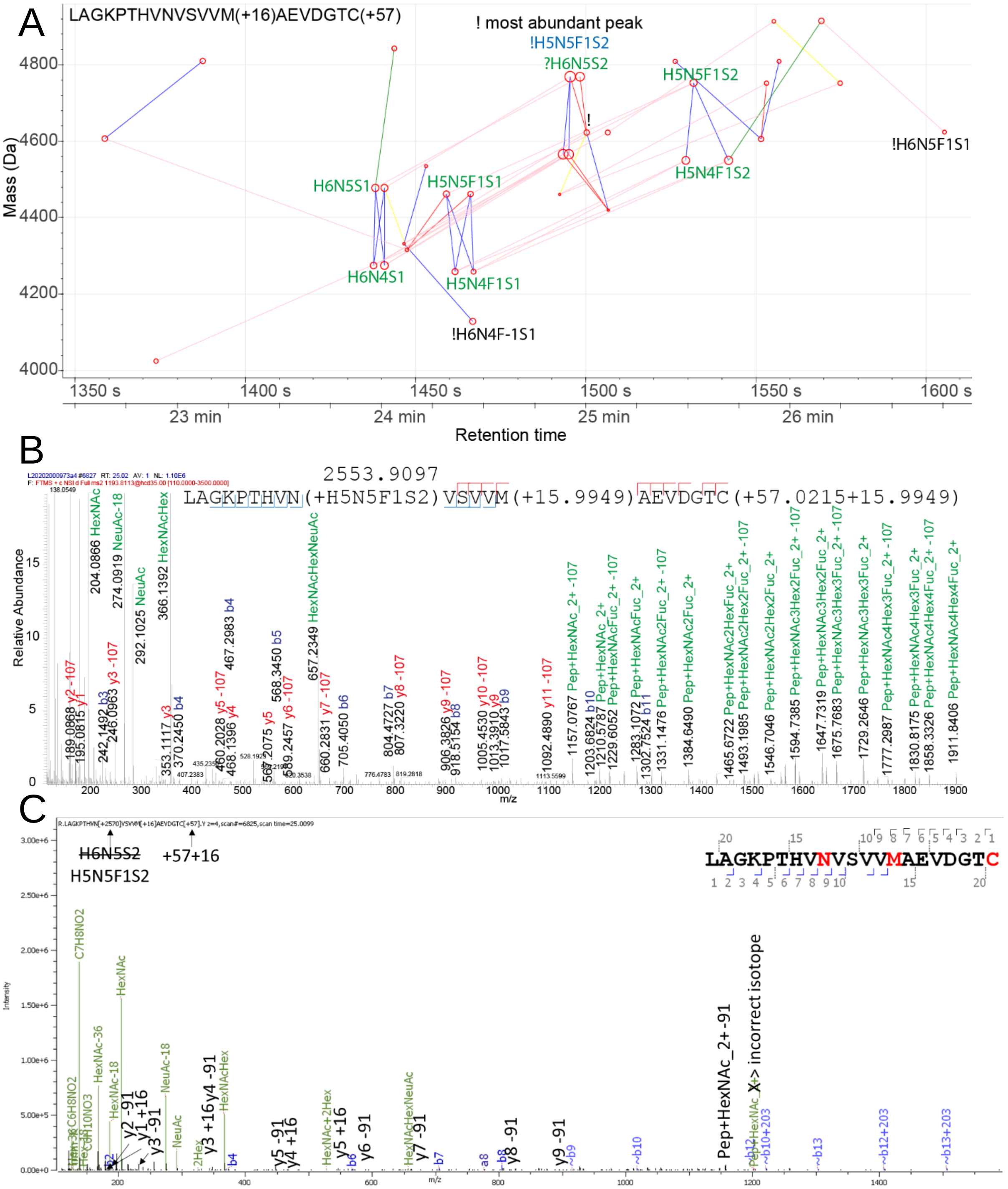

Figure S17: IgA1/2 LAGC glycopeptide cluster. (A) Representative GlycopeptideGraphMS analysis of IgA1/2 LAGC glycopeptide. Compositions in green were assigned in Byonic. (B) Manual MS/MS assignment of H5N5F1S2 glycoform with Cys oxidation*. (C) Byonic mis-assignment of LAGC glycopeptide (H6N5S2) with highest score (282). No y-ions were automatically assigned and for the Pep+HexNAc_2+, the incorrect isotope was assigned. Representative y- and Y-ions for the more likely glycoform (H5N5F1S2) with Cys oxidation (+16) and neutral loss (-91) were manually assigned (black). The LAGY glycopeptide covering the same glycosylation as LAGC site showed also an oxidized Cys (data not shown). Line colors (A): Yellow (Hex), Blue (HexNAc), Green (HexNAcHex), Red (Fucose), Pink (NeuAc).*The Cys oxidation rate was calculated representatively for H5N5F1S2 in Skyline (77.2% ± 0.4%) as fractions of the oxidized product of the overall intensities from the peptide with oxidized Cys and the expected cysteine containing peptides based on peak areas of extracted ion chromatograms in Skyline (MS1).

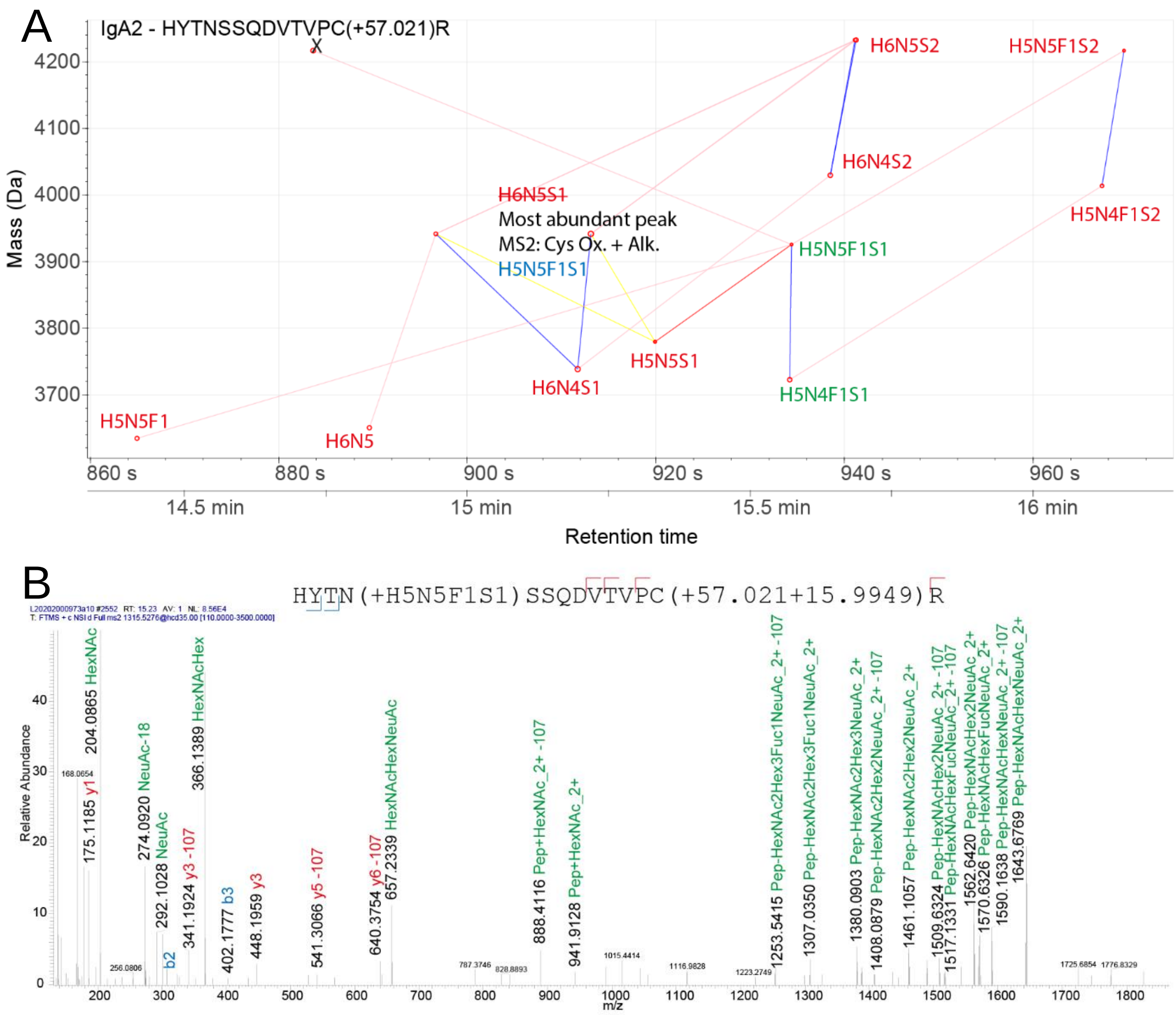

Figure S18: IgA2 HYT glycopeptide cluster. (A) Representative GlycopeptideGraphMS analysis of IgA2 HYT glycopeptide. Compositions assigned by Byonic (green), GlycopeptideGraphMS (red) or manual (blue). (B) Manual MS/MS assignment of H5N5F1S1 glycoform with Cys oxidation*. Line colors (A): Yellow (Hex), Blue (HexNAc), Green (HexNAcHex), Red (Fucose), Pink (NeuAc).

*The Cys oxidation rate was calculated representatively for H5N5F1S1 in Skyline (73.7% ± 1.1%) as fractions of the oxidized product of the overall intensities from the peptide with oxidized Cys and the expected cysteine containing peptides based on peak areas of extracted ion chromatograms in Skyline (MS1).

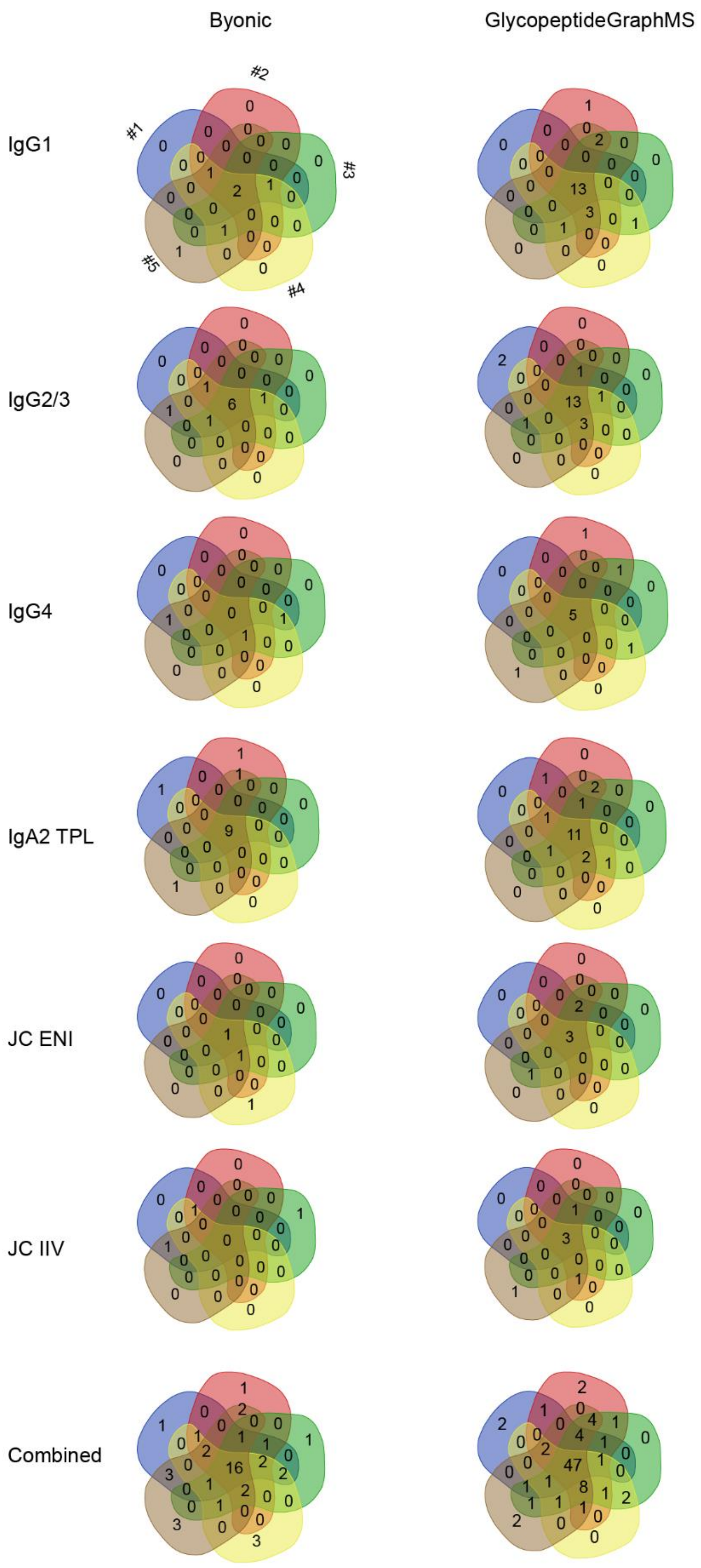

Figure S19: Evaluation of MS1 based and MS2 based glycopeptide identification consistency. Venn diagram showing glycopeptide identification consistency of 5 MS/MS (left, Byonic) and 5 MS1 only measurements (right, GlycopeptideGraphMS) for each glycopeptide of interest. False-positive identifications were excluded as described in the text. Venn diagrams were created using a web tool: http://bioinformatics.psb.ugent.be/webtools/Venn/

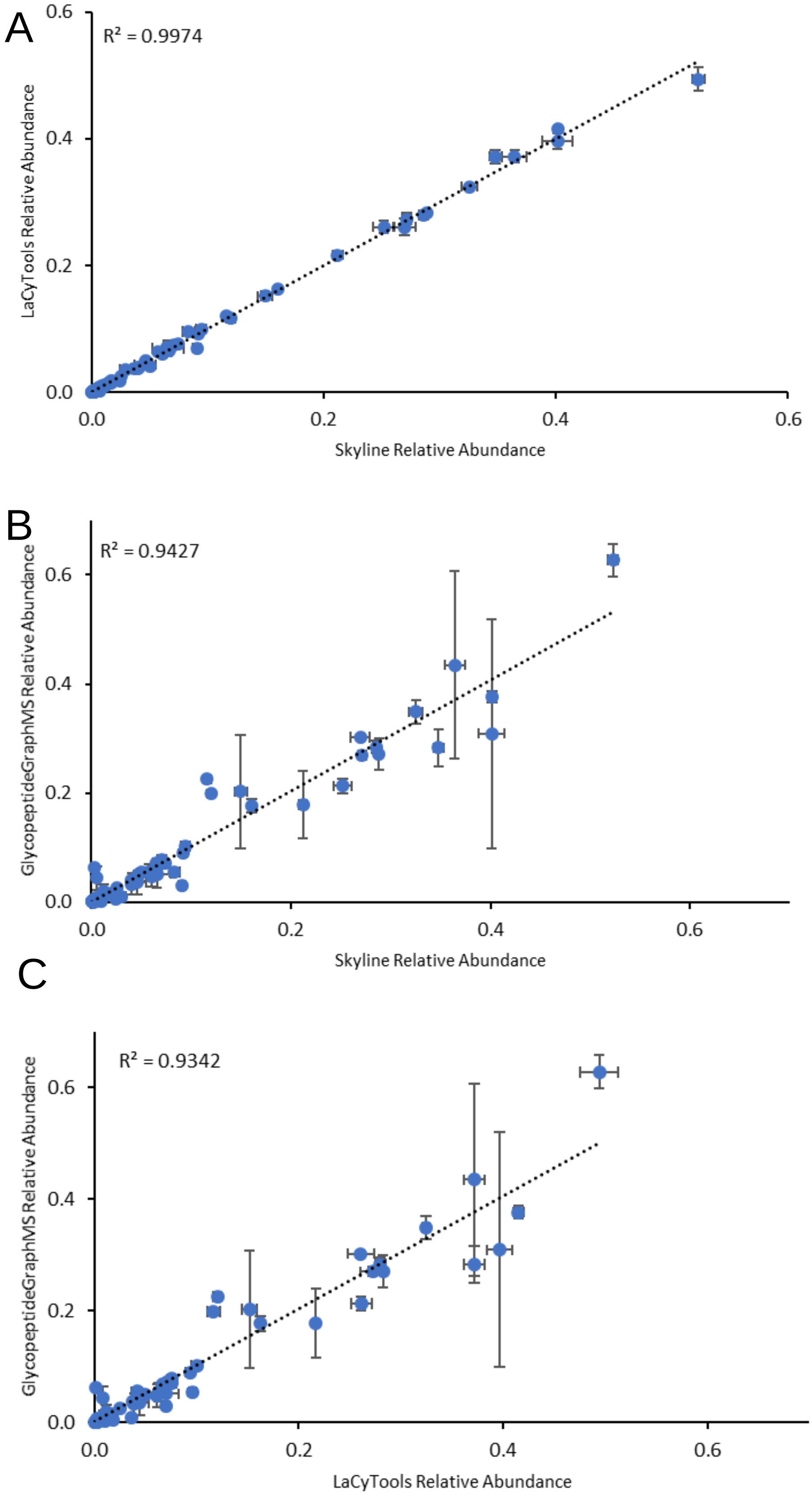

Figure S20: Similarity assessment of different quantification tools. Relative quantification (total area normalization) of (A) LaCyTools vs. Skyline, (B) GlycopeptideGraphMS vs. Skyline and (C) GlycopeptideGraphMS vs. LaCyTools. The information from all six glycopeptides clusters of interest was combined.

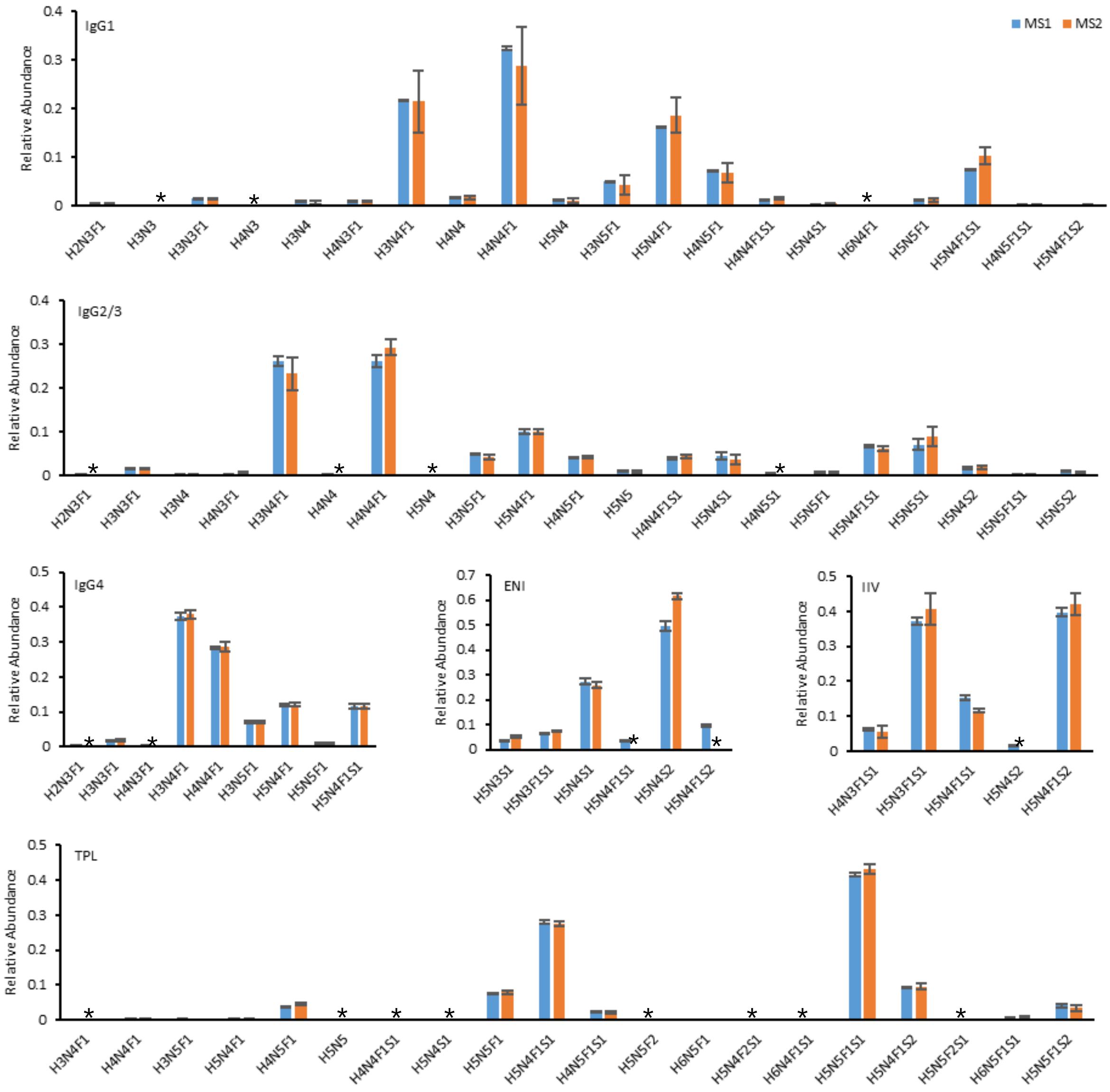

Figure S21: Relative quantification results of MS1 vs. MS2 in LaCyTools.
* did not pass the analyte curation in the MS2 data (LaCyTools)

References

1. Momčilović, A.; de Haan, N.; Hipgrave Ederveen, A. L.; Bondt, A.; Koeleman, C. A. M.; Falck, D.; de Neef, L. A.; Mesker, W. E.; Tollenaar, R.; de Ru, A.; van Veelen, P.; Wuhrer, M.; Dotz, V., *Anal. Chem.* **2020,** *92* (6), 4518-4526.
2. Ohyama, Y.; Yamaguchi, H.; Nakajima, K.; Mizuno, T.; Fukamachi, Y.; Yokoi, Y.; Tsuboi, N.; Inaguma, D.; Hasegawa, M.; Renfrow, M. B.; Novak, J.; Yuzawa, Y.; Takahashi, K., *Sci Rep.* **2020,** *10* (1), 671.
3. Lee, L. Y.; Moh, E. S. X.; Parker, B. L.; Bern, M.; Packer, N. H.; Thaysen-Andersen, M., *J. Proteome Res.* **2016,** *15* (10), 3904-3915.
